# Cross-Linking Ligation and Sequencing of Hybrids (qCLASH) Reveals an Unpredicted miRNA Targetome in Melanoma Cells

**DOI:** 10.3390/cancers13051096

**Published:** 2021-03-04

**Authors:** Ines Kozar, Demetra Philippidou, Christiane Margue, Lauren A. Gay, Rolf Renne, Stephanie Kreis

**Affiliations:** 1 Department of Life Sciences and Medicine, University of Luxembourg, 6, Avenue du Swing, L-4367 Belvaux, Luxembourg; ines.kozar@uni.lu (I.K.); demetra.philippidou@uni.lu (D.P.); christiane.margue@uni.lu (C.M.); 2 Department of Molecular Genetics and Microbiology, University of Florida, 1200 Newell Drive, Gainesville, FL 32610, USA; lagay@ufl.edu (L.A.G.); rrenne@ufl.edu (R.R.)

**Keywords:** melanoma, microRNA, qCLASH, targetome, BRAF

## Abstract

**Simple Summary:**

miRNAs are omnipresent short non-coding RNA molecules, which post-transcriptionally fine-tune the expression of most protein-coding genes in health and disease. In cancer, miRNAs have been described to directly regulate the amounts of targeted tumor suppressors and oncogenes. Although their canonical mechanism of action is well understood, the correct prediction of miRNA target genes is still a challenge. We describe here the unbiased investigation of the miRNA targetome in cancer (melanoma) cells using a technique, which allows to physically link the miRNA to its target gene. The herein identified miRNA-target interactions reveal further layers of complexity of post-transcriptional gene regulation in cancer cells and shed new light on miRNA-target gene interactions.

**Abstract:**

MicroRNAs are key post-transcriptional gene regulators often displaying aberrant expression patterns in cancer. As microRNAs are promising disease-associated biomarkers and modulators of responsiveness to anti-cancer therapies, a solid understanding of their targetome is crucial. Despite enormous research efforts, the success rates of available tools to reliably predict microRNAs (miRNA)-target interactions remains limited. To investigate the disease-associated miRNA targetome, we have applied modified cross-linking ligation and sequencing of hybrids (qCLASH) to BRAF-mutant melanoma cells. The resulting RNA-RNA hybrid molecules provide a comprehensive and unbiased snapshot of direct miRNA-target interactions. The regulatory effects on selected miRNA target genes in predicted vs. non-predicted binding regions was validated by miRNA mimic experiments. Most miRNA–target interactions deviate from the central dogma of miRNA targeting up to 60% interactions occur via non-canonical seed pairing with a strong contribution of the 3′ miRNA sequence, and over 50% display a clear bias towards the coding sequence of mRNAs. miRNAs targeting the coding sequence can directly reduce gene expression (miR-34a/CD68), while the majority of non-canonical miRNA interactions appear to have roles beyond target gene suppression (miR-100/AXL). Additionally, non-mRNA targets of miRNAs (lncRNAs) whose interactions mainly occur via non-canonical binding were identified in melanoma. This first application of CLASH sequencing to cancer cells identified over 8 K distinct miRNA–target interactions in melanoma cells. Our data highlight the importance non-canonical interactions, revealing further layers of complexity of post-transcriptional gene regulation in melanoma, thus expanding the pool of miRNA–target interactions, which have so far been omitted in the cancer field.

## 1. Background

**Figure 1:**
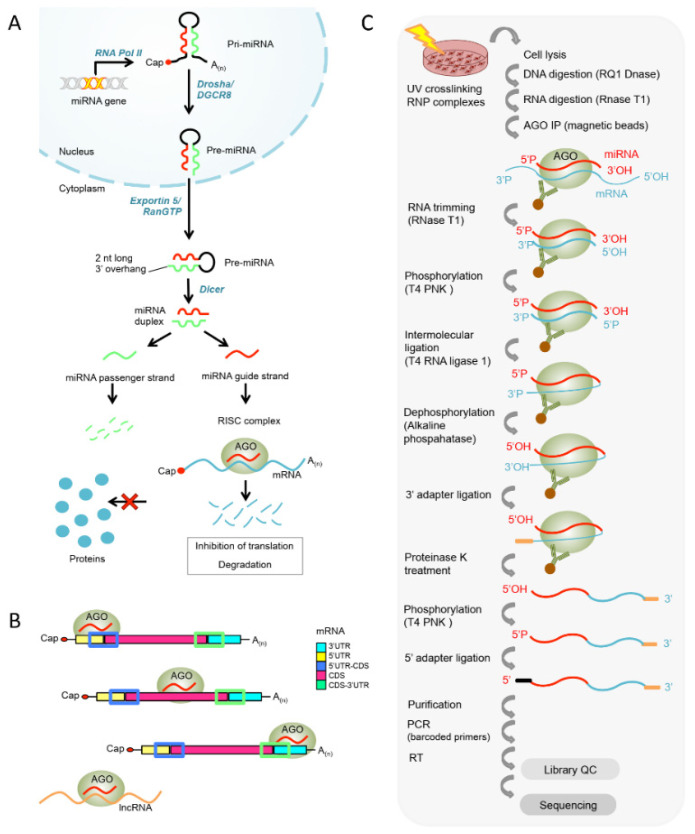
Canonical miRNA biogenesis pathway and overview of the qCLASH pipeline. (**A**) miRNAs are transcribed into a pri-miRNA by RNA polymerase II (RNA Pol II) in the nucleus. The pri-miRNA is processed by the endonuclease Drosha/DGCR8 into a pre-miRNA hairpin. The ~70 nt long pre-miRNA hairpin is exported via Exportin 5/Ran-GTP to the cytoplasm, where it is further processed by the endonuclease Dicer into a ~22 nt short miRNA duplex. One of the two miRNA strands is incorporated into the RNA-induced silencing complex (RISC) that is composed of Argonaute (AGO) and other proteins, which interact with the miRNA. Although both strands within the miRNA duplex can modulate gene expression, in most cases, the main strand is loaded onto the RISC, whereas the passenger strand is rapidly degraded due to its low half-life [25]. After binding of the miRNA to the target gene, the silencing machinery recruits the adaptor protein GW182 that directly interacts with the poly A binding protein (PABP), which in turn leads to the recruitment of the deadenylase complex, thus facilitating poly A tail shortening and the inhibition of translation initiation. Once the poly A tail is shortened, the mRNA is decapped and the destabilized target mRNA molecule is degraded. (**B**) Possible miRNA binding sites on the mRNA, namely, 5′ and 3′ untranslated regions (5′UTR and 3′UTR), coding DNA sequence (CDS), 5′-UTR-CDS, and CDS-3′UTR. Next to the mRNA, miRNAs have the potential to bind to lncRNAs and any other RNA molecules. (**C**) In the quick cross-linking ligation and sequencing of hybrids (qCLASH) protocol, RNA-protein (RNP) complexes of interest are UV cross-linked in living cells. The protein of interest is purified by immunoprecipitation (in this case, AGO bound to the miRNA and the target gene). The two interacting RNA molecules (e.g., miRNA-mRNA) are physically bound to each other by intermolecular RNA-RNA ligation, followed by library preparation and sequencing.

MicroRNAs (miRNAs) are short non-coding RNA molecules and important regulators of post-transcriptional gene expression and as such they balance gene levels, which is vital for many physiological responses to stimuli in healthy and diseased cells [1,2]. In animals, miRNAs are transcribed by RNA Pol II and further processed by several endonucleases in the nucleus (i.e., Drosha) and the cytoplasm (i.e., Dicer) into mature miRNAs, which direct the RNA-induced silencing complex (RISC) [3,4,5] to a target mRNA resulting in its degradation or the inhibition of its translation into protein [6] (Figure 1A). While the biogenesis pathway and its overall regulation as well as canonical targeting rules of miRNAs are well understood [1,6,7], we still lack insights into disease- and cell-specific miRNA–target interactions and how these regulatory networks could be exploited in therapies against complex diseases such as cancer. Many large-scale analyses have revealed differentially expressed miRNAs in cancer and especially in melanoma cells [8,9] where disturbed miRNA expression levels have been associated with disease progression and development of resistance to common therapeutic options [10,11,12,13]. While disease-specific miRNAs can easily be detected, their functional effects and mechanisms of action often remain elusive. The most common approach to date begins with screening of miRNA targets predicted by using in silico tools [14], which employ algorithms that are based on the “central dogma” of miRNA targeting: miRNAs bind via their seed sequence, which is defined as the region between nucleotides 2–7 at the 5′ end of the miRNA, to a complementary sequence in the 3′ untranslated region (3′UTR) of the mRNA (miRNA recognition element, MRE). While many methods exist that study RNA–protein interactions [15,16], techniques that allow for investigation of direct RNA–RNA interactions have only recently been developed [17,18,19]. Subsequently, these approaches have revealed that miRNAs can modulate the expression of target mRNAs by following non-predicted and non-canonical interaction patterns, for instance by binding via nucleotides beyond the seed sequence [17,18,20]. Thus, miRNAs do not only have the potential to bind regions on the mRNA other than the 3′UTR, but they can also interact with other RNA species, such as long non-coding RNAs (lncRNA) [21,22,23] (Figure 1B). MiRNAs are involved in the fine-tuning of most biological processes and have consequently been assigned functional roles in the emergence and progression of cancer cells. However, to further exploit miRNAs as therapeutic targets or as biomarkers, a more detailed understanding of their gene regulatory networks in physiological conditions is required and how these are re-wired in malignancies [9,24].

The first attempts to study large scale RNA–protein interactions were based on crosslinking proteins to RNA followed by immunoprecipitation of the protein of interest and subsequent sequencing of bound RNAs (CLIP) [26,27,28]. These three steps are the basis of all CLIP-derived techniques.

Apart from high throughput ribonomics techniques, miRNA–target interactions are generally studied on a one-by-one basis for each individual miRNA, employing time-consuming reporter gene assays, target cleavage assays, and qPCRs combined with western blots to establish down-regulation of targeted mRNAs and/or proteins [15]. The first CLIP study investigating Ago-bound miRNAs and mRNAs was Ago-HITS-CLIP [29], which was a major progress in miRNA targetome analysis. Further variants and refinements of the CLIP technology include high-throughput sequencing (HITS)-CLIP [30] and photoactivatable-ribonucleoside-enhanced (PAR)-CLIP [31]). All these techniques generate separate libraries for Ago-bound miRNAs and mRNAs resulting in two distinct datasets, which need to be bioinformatically combined. CLASH (cross-linking ligation and sequencing of hybrids), first published by the Tollervey lab in 2014 offered an elegant solution to this problem by introducing an intermolecular ligation step, which allows to physically connect the miRNA to its bound target RNA. This offers the possibility to determine real miRNA–target interactions without relying on predictions [17,32,33]. Recently, CLASH-based techniques were applied to study miRNA targetomes in different in vitro models ranging from bacterial small RNAs (RIL-seq [19]) to viral miRNAs in mammals (qCLASH [18]).

We have previously shown that the expression levels of several miRNAs display a strong co-expression with genes, which have however not been predicted to be their respective targets [8]. In order to further dissect the potentially non-canonical and non-predicted miRNA targets in melanoma, we have applied a modified cross-linking ligation and sequencing of hybrids (qCLASH) method for the first time in cancer cells [18,20]. The qCLASH technique consists of cross-linking RNA-protein (RNP) complexes in living cells, purification of the protein of interest together with the interacting RNA molecules (e.g., AGO, miRNAs, mRNAs), intermolecular RNA-RNA ligation, library preparation, and sequencing of hybrids (Figure 1C). Our qCLASH data revealed non-canonical miRNA–target gene interactions that were not predicted by commonly used in silico prediction algorithms. Interestingly, we found that miRNAs in melanoma primarily and preferentially bind the target mRNA at the coding DNA sequence (CDS), rather than the 3′UTR. Additionally, melanoma miRNAs bound some mRNAs in a non-canonical manner via regions other than the seed sequence involving the 3′ sequence of the miRNA as was previously observed in other cell models [18,34,35]. Moreover, the melanoma qCLASH dataset revealed additional RNA species that were targeted by miRNAs, such as long non-coding RNAs (lncRNAs) and primary miRNA transcripts, thus providing an unbiased overview of the miRNA targetome.

Using qCLASH, novel miRNA–target gene interactions were characterized that deviate from the central dogma of miRNA–target interactions. However, among the selected miRNA-mRNA pairs, only a small fraction of the interacting target mRNAs were repressed following miRNA transfection, suggesting that the functional consequences of miRNA–mRNA interactions might go beyond destabilization and degradation of miRNA targets. Finally, we demonstrate that ribonomics-based techniques can efficiently be used to study miRNA-mediated mechanisms in cancer and to further complete the atlas of high-confidence miRNA–target interactions.

## 2. Methods

### 2.1. Cell Lines and Cell Culture

The BRAF-mutant melanoma cell line A375 and IGR37 were purchased from ATCC and DSMZ, respectively, and 501 Mel was obtained from Dr. Ruth Halaban (Dermatology department, Yale School of Medicine, USA). The BRAF inhibitor-resistant melanoma cell line A375_XP was generated as described in Kozar et al., 2017 [8]. The miR-509 expressing cell line A375_miR509 was generated by lentiviral transduction of pre-miR509-1 (Biosettia, San Diego, CA, USA). All melanoma cell lines were cultured in RPMI 1640 medium containing ultraglutamine (Lonza BioWhittaker, Basel, Switzerland), supplemented with 10% FCS (Fetal Calf Serum, GIBCO ThermoFisher Scientific, Gent, Belgium) and 1% PS (10,000 U/mL Penicillin and 10,000 U/mL Streptomycin, Lonza BioWhittaker, Basel, Switzerland) and grown at 37 °C in a humidified atmosphere at 5% CO_2_. Cells were regularly tested to be mycoplasma-free.

### 2.2. qCLASH

Briefly, 5 × 10^7^ cells in biological triplicates were UV cross-linked at a wavelength of 250 nm and 600 J/cm^2^. Argonaute (Ago) protein immunoprecipitation was performed using Protein G Dynabeads (Invitrogen, ThermoFisher Scientific, Gent, Belgium, cat. number 10004D), and 10 μL 2A8 anti-Ago antibody (Millipore, Overijse, Belgium, cat. number MABE56) per sample. qCLASH sample preparations were performed exactly as previously described by Gay et al., 2018 [18] and as illustrated in Figure 1.

### 2.3. Sequencing and Bioinformatic Analyses

qCLASH libraries were sequenced on a HiSeq3000 instrument with a read length of 100 bp. The raw sequences were pre-processed and analyzed as described by Gay et al., 2018 [18], using the bioinformatics pipeline Hyb [36], and custom scripts available at GitHub (https://github.com/RenneLab/qCLASH-Analysis, accessed on 3 March 2021). The miRNA-mRNA-lncRNA networks were generated using the tidygraph R package (https://github.com/thomasp85/tidygraph, accessed on 3 March 2021). The TCGA expression data was downloaded and analyzed using the TCGAbiolinks R/Bioconductor package (https://bioconductor.org/packages/release/bioc/html/TCGAbiolinks.html, accessed on 3 March 2021).

### 2.4. Mimic Transfections

A total of 50,000 cells were transfected in 12-well plates with 25 nM and 50 nM miRNA mimic for 48 h and 72 h. Negative control mimic (NCM) (Qiagen, Venlo, The Netherlands, cat. number YM00479902-ADA), miR-100-5p (Qiagen, Venlo, The Netherlands, cat. number YM00472477-ADA), miR-34a-5p (Qiagen, Venlo, The Netherlands, cat. number YM00473212-ADA), miR-320a-3p (Qiagen, Venlo, The Netherlands, cat. number YM00471432-ADA), let-7a-5p (Qiagen, Venlo, The Netherlands, cat. number YM00470408-ADA) miRCURY LNA miRNA mimics were purchased from Qiagen, Venlo, The Netherlands and transfected using the HiPerfect transfection reagent (Qiagen, Venlo, The Netherlands, cat. number 301707) according to the manufacturer’s instructions. The mimic transfections were performed in biological duplicates and technical triplicates.

### 2.5. RNA Extraction

Total RNA was extracted in biological replicates each consisting of three technical replicates using the Quick-RNA^TM^ Mini- Prep Kit (ZYMO Research Corp, Irvine, CA, USA), following the manufacturer’s instructions. Cells were collected from triplicates and lysed in 300 μL RNA-Lysis Buffer. RNA purity and quantity were assessed using the NanoDrop2000 Spectrophotometer (Isogen Life Science, Utrecht, The Netherlands).

### 2.6. Quantitative PCR

Total RNA was reverse-transcribed using the miScript RT II kit (Qiagen, Venlo, Netherlands, cat. number 218161) in a total reaction volume of 10 μL, according to the manufacturer’s instructions. Quantitative real time PCR (qPCR) was carried out on a CFX384 Detection System (Bio-Rad, Hercules, CA, USA) in a total volume of 10 μL (10 pmol of each primer and cDNA corresponding to 5 ng and 50 ng RNA template for miRNA and mRNA, respectively). The reference miRNAs (RNU1A, RNU5A, SCARNA17) and genes (HPRT, TBP, PPIA), as well as the target miRNAs and genes were assayed in parallel for each sample. All samples were run in biological duplicates each composed of technical triplicates. miRNA-specific primers were purchased from Qiagen (Venlo, The Netherlands) and qPCR primers for gene amplification were purchased from Eurogentec (Liège, Belgium). Gene amplification qPCR primers used herein (5′→3′): HPRT (F: TGGACAGGACTGAACGTCTT; R: GAGCACACAGAGGGCTACAA), TBP (F: ACCCAGCAGCATCACTGTT; R: CGCTGGAACTCGTCTCACTA), PPIA (F: CAGACAAGGTCCCAAAGACA; R: CCATTATGGCGTGTGAAGTC), AXL (F: GAGACCCGTTATGGAGAAGTGT; R: 5′ CTGATGCCCAGGCTGTTCAAG), CD68 (F: GAGACCATTGGAGACTAC; R: GGGTTCAGTACAGAGATG), FOSL1 (F: CCTTGTGAACAGATCAGC, R: TCCAGTTTGTCAGTCTCC), CIZ1 (F: CCAGCAAAGAGATTGAGG, R: GGTGTCTGTGTCTGTTTC), INTS3 (F: CAAGAGACAGAGCCTGAGGA; R: GTTCTGCTTGGTGTTGGTCA), MDM2 (F: GTGAGGAGCAGGCAAATG; R: GGTCTAACCAGGGTCTCTT), MDM4(F: CTCCGTGAAAGACCCAAG; R: CTGAGCAGCATCTGTAGTAG), SERPINE2 (F: CATCAGCACCAAGACCATAG; R: CAGCGGCTCCTTCAAATC), ERBB3 (F: GCTGAGATAGTGGTGAAGG; R: CACTGAGGAGCACAGATG), PKM2 (F: CGTGGATGATGGGCTTAT; R: AGGTTCACACCCTTCTTG), RPS4X (F: GGAGACTGGCAAGATTACTG; R: CACGTGAACCACGTCAAA), RPS6KA3 (F: GGACCAACTGCCACAATAC, R: CCGCTGAGCAAGAGTAGA), RPS6 (F: GTCCGCCAGTATGTTGTAAG; R: CTTGGTACGCTGCTTCTTC), MYC (F: CGACTCTGAGGAGGAACA; R: GTGAGGAGGTTTGCTGTG), SPRY2 (F: TGCCATCAGACTGGATCT; R: CAGCACAGTTGTCCTCATC), SPRY4 (F: GCCTGTGGGAAGTGTAAA; R: GTGCCATAGTTGACCAGAG), LRP8 (F: GTCACTGCCGCTGTTATC; R: CTTCCGCTTCCAGTTTCTC), RHOBTB3 (F: TTCAAGGTACGACAGTGCCA: R: ACGGGAATCAGGACACTCTT), TUCS2(F: GGATCTGGCTCACGAGTTCT; R: TTCACGATGCCCTGAGGAAT), NF1 (F: ATAAGCCCTCACAACAACCA; R: ACTCGGTGCCATTCGTATT), DICER1 (F: GAAAATATCAGGTTGAACTGCTTG; R: GATAGGACAGCTCTTTAGTGAGTAGTAC), JUNB (F: GGAACAGCCCTTCTACCAC; R: TCGGTTTCAGGAGTTTGTAG). AGO1, AGO2, AGO3, AGO4-specific primer sequences can be found in Völler et al., 2016 [37]. The geometric mean of three reference genes was calculated and a normalization factor for each sample was generated using geNorm (VBA add-in for Microsoft Excel) or using the BioRad (Hercules, CA, USA) CFX Manager Software. The normalization factor was used to calculate the relative amount of each target miRNA and mRNA in each sample.

### 2.7. Statistical Analysis

Statistical significance was determined by one-way ANOVA, followed by Dunnett’s multiple comparisons test using GraphPad Prism (San Diego, CA, USA) Version 8.0.2. ns, not significant; * *p* ≤ 0.05; ** *p* ≤ 0.01; *** *p* ≤ 0.001; **** *p* ≤ 0.0001.

### 2.8. Western Blot

Cells were lysed at 4 °C using ice cold lysis buffer containing 30 mM Tris/HCl pH 6.7, 5% glycerol, 2.5% β-mercaptoethanol, and 1% SDS. Protein extracts were analyzed by SDS-PAGE and western blotting. Enhanced chemiluminescence (ECL) signals were detected as described before [38]. The following antibodies were used for western blot: anti-AXL C89E7 (Cell Signaling Technology, Boston, MA, USA), anti-CD68 (Cell Signaling Technology, Boston, MA, USA), anti-DICER1 clone D38E7 (Cell Signaling Technology, Boston, MA, USA), anti-SPRY4 (GeneTex, Irvine, CA, USA), pan-AGO clone 2A8 (Millipore, Overijse, Belgium), anti-vinculin E1E9V XP (Cell Signalling Technology, Boston, MA, USA), anti-alpha-tubulin (Santa Cruz, Heidelberg, Germany). HRP-labelled secondary antibodies were purchased from Cell Signaling Technology (Boston, MA, USA).

### 2.9. Dual Luciferase Reporter Gene Assays

Partial sequences of AXL, CD68, MDM4, DICER1, and SPRY4 3′UTR and CDS containing putative let-7a-5p, miR-100-5p, miR-320a-3p, or miR-34a-5p target sites were cloned (Genecust, Boynes, France) into the pmirGLO Dual Luciferase miRNA target expression vector (Promega, Leiden, Netherlands) downstream of the firefly luciferase gene. The day prior transfection, A375 cells were seeded at a density of 25 × 10^3^–50 × 10^3^ cells/well in a 24-well plate. Cells were transiently transfected with 500 ng plasmid and 50 nM miRNA mimic or negative control for 24 h, 48 h, or 72 h using Dharmafect Duo (Thermo Scientific, Gent, Belgium). Samples were lysed with 1× Passive Lysis Buffer (Promega, Leiden, Netherlands) and luciferase activities were measured consecutively according to the manufacturer’s instructions using the Cytation 5 Cell Imaging Multi-Mode Reader (BioSPX B.V., Biotek, Highland Park, TX, USA). The Firefly/Renilla activity ratios of mimic-treated samples were calculated and normalized to the respective ratios of the negative control-treated samples for each construct and each time point. Statistical significance was determined by one-way ANOVA, followed by Dunnett’s multiple comparisons test using GraphPad Prism Version 8.0.2. ns, not significant; * *p* ≤ 0.05; ** *p* ≤ 0.01; *** *p* ≤ 0.001; **** *p* ≤ 0.0001.

## 3. Results

In a previous study, we have investigated miRNome and transcriptome changes in melanoma cells resistant to targeted BRAF inhibition therapy, followed by a miRNA-mRNA co-expression analysis with the aim to identify miRNA–target interactions potentially involved in the emergence and maintenance of drug resistance [8]. As the majority of co-expressed pairs had not been predicted to interact, we applied qCLASH, which allows for unbiased identification of high-confidence miRNA–target interactions irrespective of binding characteristics of the interacting molecules (Tables S1 and S2). In qCLASH, RNA-protein (RNP) complexes are UV cross-linked, the protein of interest (e.g., Argonaute) is purified together with the bound RNA molecules (e.g., miRNAs and mRNAs), which are covalently attached to each other during an intermolecular RNA-RNA ligation step, followed by library preparation and sequencing of the hybrid molecules (Figure 1C).

### 3.1. Characteristics of miRNA Hybrids Identified in Melanoma Cells

**Figure 2:**
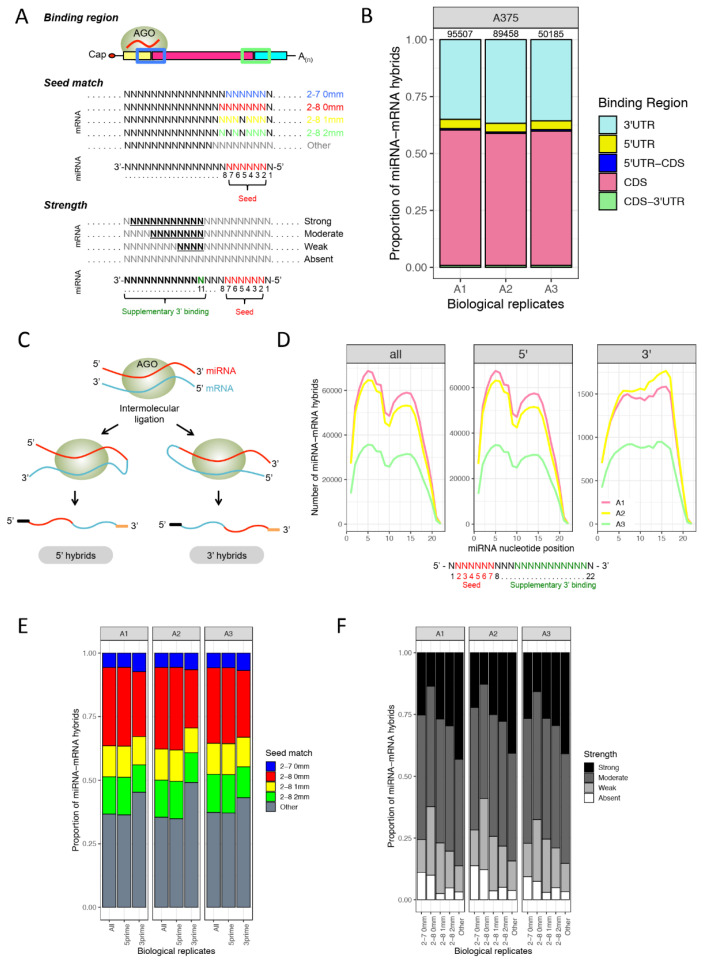
Characteristics of miRNA-mRNA hybrids discovered in melanoma cells. (**A**) Binding characteristics of miRNA–mRNA interactions. Binding region: mRNA part of the hybrids mapped to the ENSEMBL transcript database with regions corresponding to the 5′UTR and 3′UTR, CDS, 5′-UTR-CDS, and CDS-3′UTR. Seed match: nucleotides (nt) 2–7 at 5′ end of miRNA with zero (2–7, 0 mm), 2–8 with zero (2–8, 0 mm), one (2–8, 1 mm), or two mismatches (2–8, 2 mm), and the remaining binding modes were classified as “other”. Seed pairing at nucleotides 2–7 (blue) and 2–8 (red) without mismatches was considered as canonical seed pairing, whereas any additional type of pairing was referred to as non-canonical seed pairing. Strength: “strength” of binding outside the seed region at the 3′ end of the miRNA based on number of bound nt: >8 nt (strong), 5–8 nt (moderate), 1–4 nt (weak), and 0 nt (absent). (**B**) Proportion of miRNA-mRNA hybrids across three biological replicates of the BRAF-mutant melanoma cell line A375 (A1, A2, and A3) with miRNA binding sites mapped to the mRNA sequence, and the number of identified hybrid sequences in each replicate. Hybrid mRNAs for which the transcript was not annotated in the corresponding database were excluded. The number above the bars displays the number of hybrids identified in each biological replicate. (**C**) The two types of miRNA-mRNA hybrid molecules that were generated during the intermolecular ligation: 5′ and 3′ miRNA hybrids, in which the miRNA is located at the 5′ end or the 3′ end of the hybrids, respectively. (**D**) M-plots showing the number of miRNA-mRNA hybrids bound at each miRNA nt position (*x*-axis, nt 1–22), and showing that most genes are targeted via the seed sequence but also with supplementary sites in the 3′ region of the miRNA. (**E**) The type of seed pairing (5′ miRNA sequence) was divided into classes as shown in (**A**). The proportion of miRNA-mRNA hybrids across the three biological replicates was plotted for the different seed categories for all hybrids as well as for 5′ and 3′ hybrids separately. (**F**) miRNA base-pairing via the 3′ portion of the miRNA (supplementary 3′ or non-seed pairing). The strength of 3′ sequence binding was classified as in (**A**) and was plotted for each seed sequence type across the biological replicates.

The miRNA-mRNA hybrids in melanoma cells were investigated, focusing on the miRNA-bound region on the mRNA (binding region), the type of 5′ seed pairing (seed match), as well as supplementary seed pairing at the 3′ end of the miRNA (strength) (Figure 2A). The number of high confidence miRNA-mRNA hybrids discovered in the three biological replicates of the BRAF-mutant melanoma cell line A375 (A1, A2, and A3) ranged between 50 and 95 K (Figure 2B, Tables S1 and S2). In order to characterize the binding nature of these hybrids, the mRNA portion of the hybrid was mapped to the ENSEMBL transcript database. The regions were divided into 5′UTR, 5′UTR-CDS, CDS, CDS-3′UTR, and 3′UTR. Hybrid mRNAs for which the transcript was not annotated in the corresponding database were excluded. Surprisingly, in more than 60% of hybrids, the miRNA target sites were located in the CDS of the mRNA (Figure 2B). The 3′UTR region of the mRNA, on the other hand, which is primarily assessed by miRNA target prediction algorithms, accounted for only 30% of miRNA interactions (Figure 2B). These observations confirm previous findings [17,18] and highlight the fact that our current understanding of miRNA targeting the 3′UTR of mRNAs needs to be revisited.

### 3.2. miRNA 5′ Seed-Pairing and 3′ Non-Seed Pairing (Supplementary 3′ Pairing)

During the intermolecular ligation step, two types of miRNA-mRNA hybrid molecules have been generated: ~95% of hybrids were 5′ miRNA hybrids (miRNA located at 5′ end of the hybrid) and ~5% were 3′ miRNA hybrids (Figure 2C). Further analyses were performed for both hybrid types separately and for all hybrids combined. Using the Vienna diagrams created by the Hyb pipeline [36], we explored the involvement of individual miRNA nucleotides (nt) in interactions with the targets, plotting the frequency of each miRNA nt pairing with the mRNA portion of the hybrid thus providing a broad picture of intermolecular base-pairing within the dataset (Figure 2D). The majority of intermolecular interactions in all and the 5′ hybrids alone are governed by the seed region but also involve a substantial amount of supplementary nt at the 3′ end of the miRNA. Only the central region around nt 10 appears to contribute less to the miRNA binding. The small number of 3′ hybrids showed different binding patterns in which the supplementary nt at 3′ miRNA end seemed to play a more prominent role compared to the seed region, which is still under investigation (Figure 2D). Furthermore, in order to characterize the miRNA seed binding, the type of seed pairing was divided into classes consisting of canonical seed pairing with nt 2–7 at the 5′ end of the miRNA with zero mismatches (2–7, 0 mm) and nt 2–8 with zero mismatches (2–8, 0 mm), as well as non-canonical seed pairing with seed interactions with one (2–8, 1 mm) or two mismatches (2–8, 2 mm), and the remaining binding modes were classified as “other”. Overall, the canonical seed region at the 5′ end of the miRNA was bound to the mRNAs at a relatively high frequency (seed match 2–7 (blue) and 2–8 (red) with 0 mismatches) (Figure 2E). Interestingly, when looking at all and the 5′ hybrids individually, up to 60% of miRNAs did not display a canonical seed pairing, and 3′ hybrids showed a higher proportion of non-canonical seed pairing compared to 5′ hybrids (Figure 2E). Furthermore, the binding at the 3′ end of the miRNA was closely inspected and depending on the number of nt of the miRNA bound to the mRNA, it was classified as strong (>8 bound nt), moderate (5–8 bound nt), weak (1–4 bound nt), or absent (0 bound nt). Interestingly, miRNAs that tend to have non-canonical seed pairing have a higher proportion of strong binding sites at the 3′ end of the miRNA (Figure 2F). This demonstrates that although most miRNAs preferentially bind via their seed sequence, they also have additional compensatory or supplementary binding sites at the 3′ end as reported previously [1,18]. Thus, miRNAs exhibit additional binding possibilities, which allow for more extensive interactions with their target. The fact that miRNAs can bind via seed and/or 3′ regions might explain how some miRNAs with the same seed sequence can have different targets. Overall, the herein observed characteristics of miRNA–mRNA interactions were consistent with qCLASH data obtained from additional melanoma cell lines that were tested in independent biological replicates, which in addition shows that these interactions are independent of genetic and phenotypic characteristics of cancer cells. (Figure S1A–D).

### 3.3. Hybrid Frequency and miRNA Expression Levels

**Figure 3:**
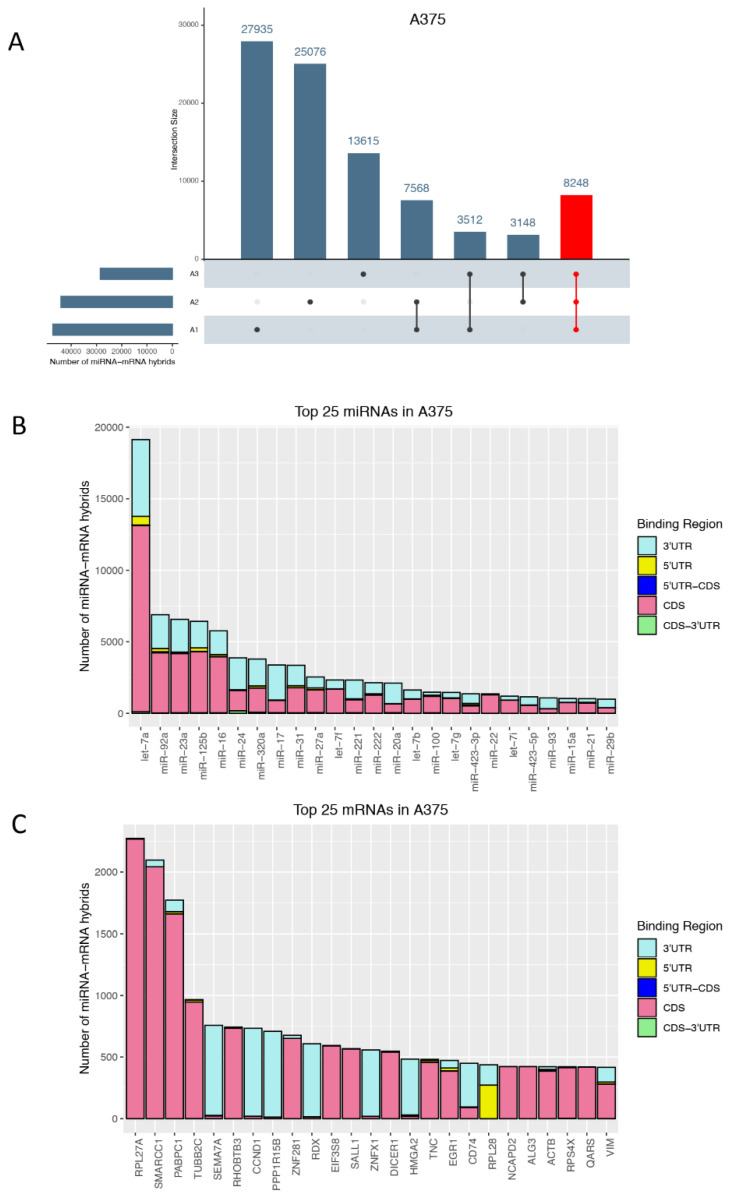
Most commonly occurring hybrids. (**A**) UpSet plot representing the number of distinct miRNA-mRNA hybrids across three biological replicates (horizontal bars) that are unique or common across the replicates (vertical bars). miRNA-mRNA hybrids present in all replicates (A1, A2, and A3) of the BRAF-mutant melanoma cell line A375 were highlighted in red. Top 25 (**B**) miRNAs and (**C**) mRNAs that occur in miRNA-mRNA hybrids showing the number of hybrids detected and the corresponding binding region on the mRNA. As opposed to the data shown in (**A**), the number of hybrids in (**B**,**C**) was not collapsed for unique miRNA-mRNA pairs; thus, it displays the number of all sequences/hybrids that were identified for that particular miRNA (**B**) or mRNA (**C**).

In a next step, we looked at miRNAs and mRNAs that occurred most frequently in qCLASH hybrids (Figure 3). For most miRNA-mRNA hybrid pairs, several different sequences were found. To establish how many miRNA-mRNA hybrid pairs were only found in one biological replicate or shared between two or all three biological replicates, the unique hybrid pairs were plotted (Figure 3A). While many hybrid pairs were identified in only one biological replicate, more than 8 K miRNA-mRNA hybrids were found in all replicates (Figure 3A). In the following analyses, we focused on common hybrids as they were considered the most consistent miRNA–mRNA interactions in the melanoma cell line A375, regardless of the nature of interaction (e.g., seed match, and binding region). Most miRNAs occurring with high frequency in hybrids seem to be able to bind to any region along the mRNA sequence with a strong preference for the CDS, followed by the 3′UTR (Figure 3B). While miRNA binding is almost uniformly distributed across the different mRNA regions (Figure 3B), certain mRNAs, on the other hand, are targeted in defined regions (Figure 3C). For instance, let-7a, present in almost 20 K hybrids can bind any region on its target mRNAs with a preference for the CDS, followed by the 3′UTR (Figure 3B). Comparable trends were observed in additional melanoma cells, where let-7a, miR-92a, and miR-125b were, for instance, also among the top miRNAs found in miRNA-mRNA hybrids (Figure S1E–G). However, when analyzing specific target mRNAs, some of them were almost exclusively targeted at the CDS (e.g., RPL27A, and SMARCC1), while others were preferentially targeted at the 3′UTR (e.g., SEMA7A, and CCND1), with very few exceptions such as RPL28 that was targeted at both the 3′ and 5′ UTR (Figure 3C).

**Figure 4:**
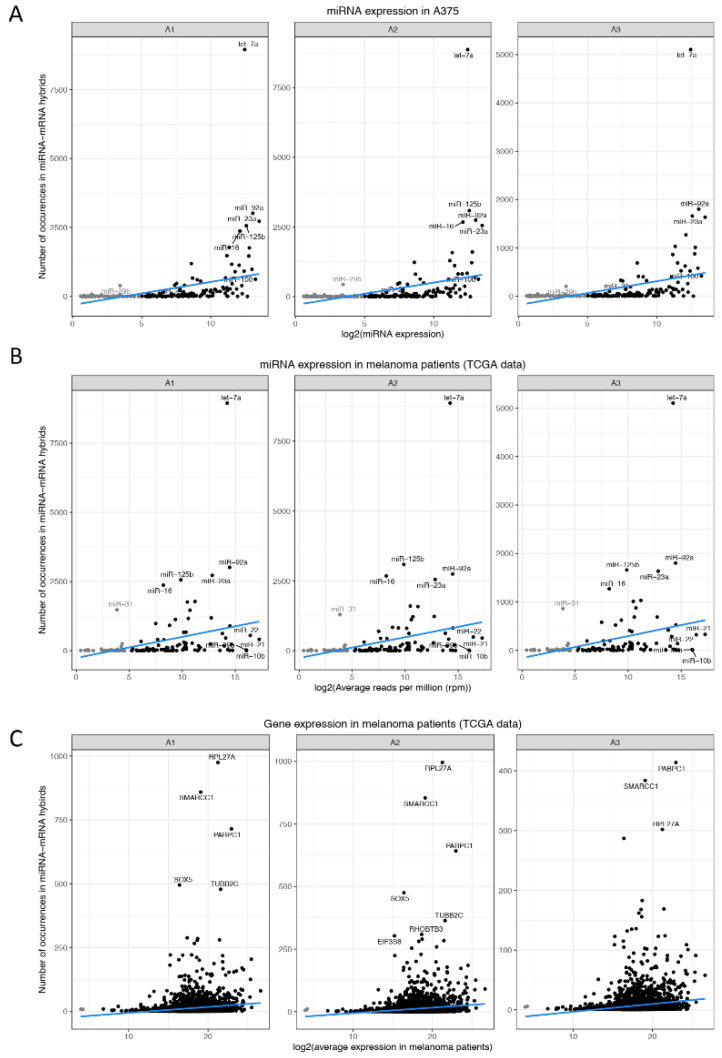
Correlation between hybrid number and miRNA or mRNA expression levels. Number of miRNA occurrence frequency in miRNA-mRNA hybrids of the biological replicates (A1, A2, and A3) of the melanoma cell line A375 compared to (**A**) log2 miRNA expression levels in A375 [8] and (**B**) log2 average reads per million (rpm) in melanoma patient samples (TCGA 2015 data). (**C**) Number of mRNA occurrence frequency in miRNA-mRNA hybrids compared to log2 gene expression (mRNA) levels in melanoma patient samples (TCGA2015 data).

In order to verify whether miRNAs most commonly occurring in hybrids are more likely to ligate with their corresponding target due to their high abundance, we correlated the occurrence frequency of miRNAs to their respective expression levels that have been measured by microarrays [8] (Figure 4A) or RNAseq [39] (Figure 4B,C). While some highly abundant miRNAs in melanoma cells (e.g., let-7a, miR-92a) also frequently occur in hybrids, the majority of these miRNAs showed a modest correlation between miRNA presence within hybrids and miRNA expression levels (r ≈ 0.39). Similarly, the occurrence frequency of miRNAs (Figure 4B) and mRNAs (Figure 4C) in hybrids was also compared to expression data from melanoma patients [39] showing a correlation of r ≈ 0.3 and r ≈ 0.13, respectively (Figure 4B). Accordingly, while some miRNAs (e.g., let-7a, and miR-92a) are highly expressed and frequently appear in hybrids, others display high expression levels yet a low occurrence frequency (e.g., miR-21), or low expression levels and relatively high frequency in miRNA-mRNA hybrids (e.g., miR-31) (Figure 4B) suggesting that the presence of miRNAs in hybrids is not solely driven by their abundance and that mechanisms linked to miRNA function are more likely to play a role including miRNA stability and turnover [40,41].

**Figure 5:**
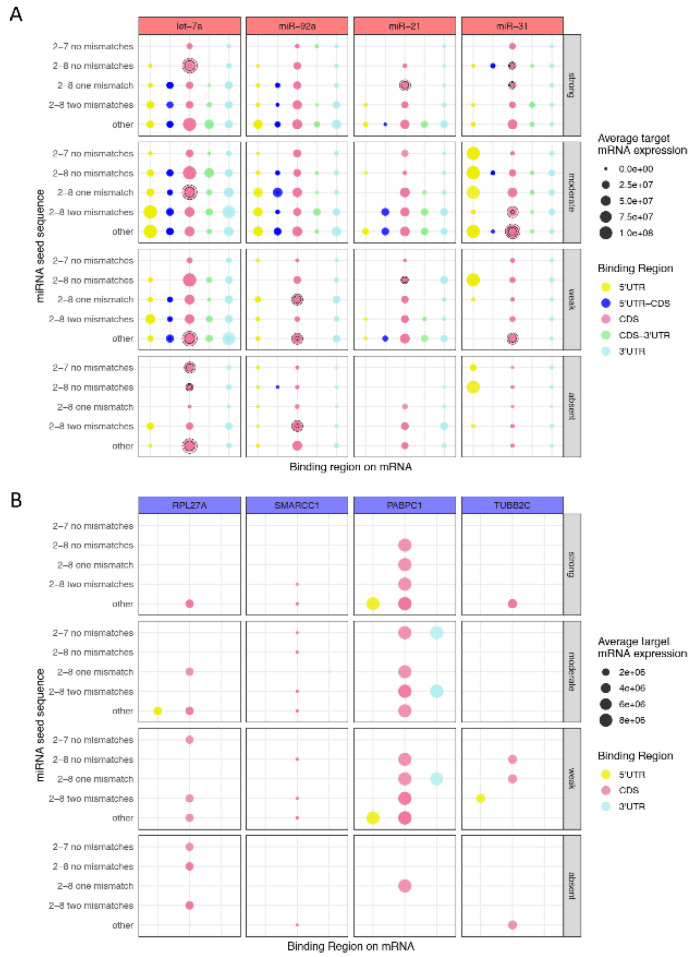
Comparison of miRNA binding characteristics with target mRNA expression levels. (**A**) miRNAs frequently occurring in hybrids and with high expression levels (let-7a, miR-92a, miR-21) or low expression levels (miR-31) in TCGA melanoma patient data (TCGA2015) were selected. The size of the circles corresponds to the average expression of a given target mRNA. For each of the four selected miRNAs, several overlapping dots are displayed (highlighted with black circles), as the miRNA can bind to several different targets. (**B**) Selected mRNAs frequently occurring in hybrids and with high expression levels in melanoma patient data (TCGA2015) (RPL27A, SMARCC1, RAPBC1, and TUBB2C) are shown. The binding characteristics (seed match, supplemental 3′ pairing or strength, and binding region on mRNA) are illustrated as well as the average target mRNA expression levels. The size of the circles represents the average mRNA expression, which remains constant, as the expression of the gene is stable. The exact values, expression levels, and binding characteristics are illustrated in Table S3.

Next, the binding characteristics of the above mentioned miRNAs (let-7a, miR-92a, miR-21, and miR-31) to their target mRNA were closely inspected by plotting the seed interactions (left *y*-axis), the strength of the supplementary binding at the 3′ end of the miRNA (right *y*-axis) and the binding region on the mRNA (*x*-axis, illustrated by the color of the dots), as well as the average expression [39] of the different target mRNAs that fall into each category (illustrated by the size of the dot) (Figure 5A; Table S3). Notably, although miRNAs have targets falling in different binding categories, the more canonical the miRNA pairing at the 5′ end and the stronger the supplementary binding at the 3′ end is, the lower the expression of the target mRNA is (Figure 5A), Table S3. In addition, mRNAs targeted at the 5′ UTR and CDS appear to have higher expression levels, whereas those that are especially targeted at the 3′UTR display lower levels, suggesting that mRNAs targeted in a canonical fashion involving their 3′UTR and the miRNA seed region are more likely to be negatively regulated (Figure 5A; Table S3). A similar analysis was performed for the top four genes occurring in miRNA-mRNA hybrids, namely ribosomal protein 27a (RPL27A), SWI/SNF Related, Matrix Associated, Actin Dependent Regulator Of Chromatin Subfamily C Member 1 (SMARCC1), Poly A binding protein cytoplasmic 1 (PABPC1), and tubulin β 2C (TUBB2C) (Figure 5B; Table S3). The expression levels of each mRNA remain unchanged in melanoma patients [39] regardless of the binding characteristics as well as the miRNA that they are targeted by. It is, however remarkable that miRNAs targeting RPL27A, PABPC1, or TUBBB2C with strong supplementary binding at the 3′ end of the miRNA and non-canonical seed pairing (“other”), usually target the mRNA in the CDS (Figure 5B; Table S3). Conversely, miRNAs that target PABPC1 at the 3′UTR tend to follow canonical seed pairing or non-canonical seed pairing with up to two mismatches and have rather weak to moderate supplementary binding at the 3′ end (Figure 5B). This indicates that miRNAs targeting mRNAs outside the 3′UTR have a higher tendency to follow non-canonical binding patterns.

### 3.4. Functional Analysis of Canonical and Non-Canonical Binding Sites

**Figure 6:**
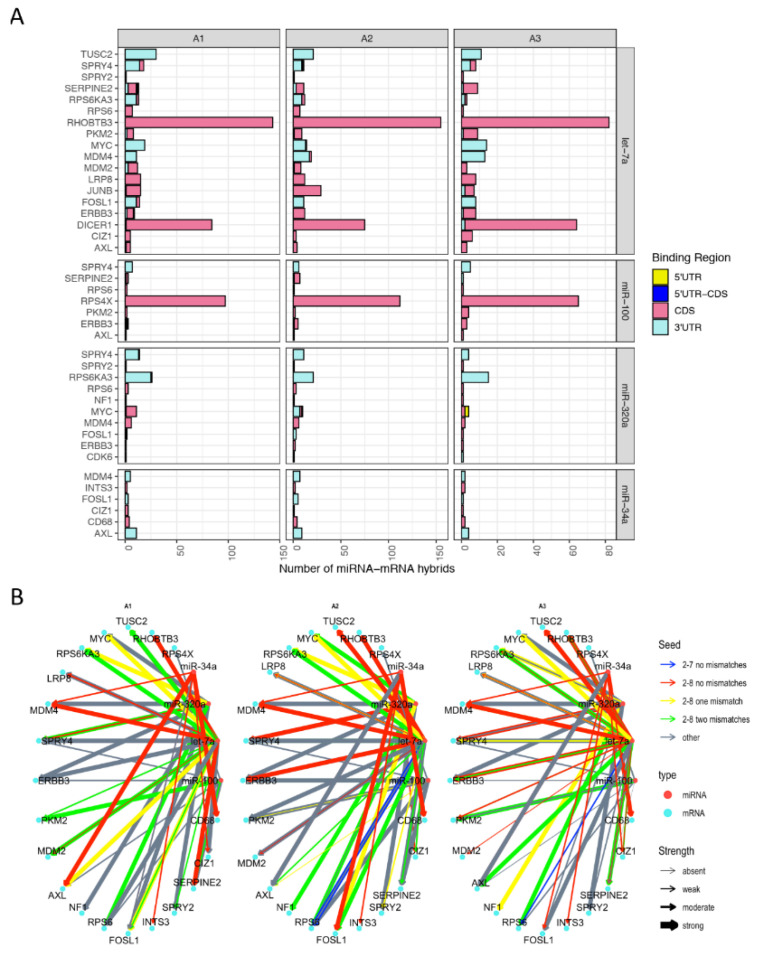
Selected miRNA-mRNA hybrids. (**A**) Selected mRNA targets (left *y*-axis) of let-7a, miR-34a, miR-100, and miR-320a (right *y*-axis) with number of occurrences (*x*-axis) and the binding region across three biological replicates. (**B**) Networks of miRNA (red dots) and mRNA (blue dots) interactions found in hybrids of A1, A2, and A3 using tidygraph. Arrow color represents the miRNA 5′ seed match and arrow thickness corresponds to the strength at the 3′ end of the miRNA.

**Figure 7:**
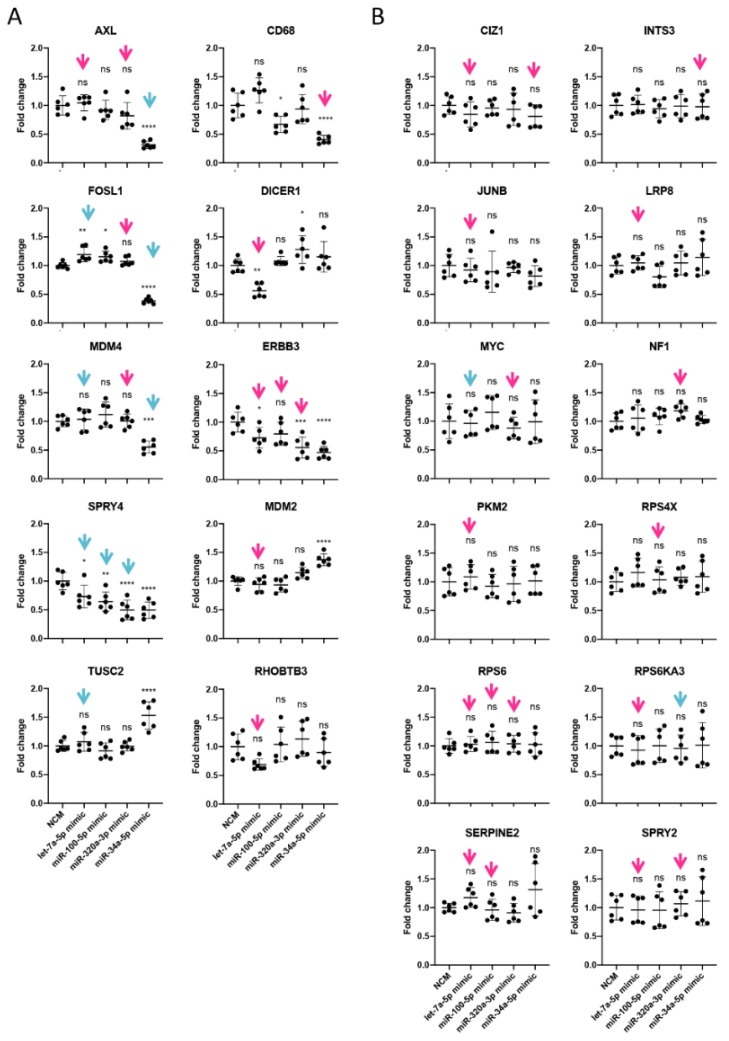
Expression of selected target mRNAs following transfection with 4 different miRNA mimics. The expression levels were normalized to the corresponding negative control mimic (NCM). Illustrated are 22 genes targeted at the 3′UTR (blue arrows) or CDS (red arrows) whose expression changes (**A**) or remains unaltered (**B**) upon 50 nM mimic treatment for 72 h. Statistical significance was determined by one-way ANOVA, followed by Dunnett’s multiple comparisons test with ns, not significant; * *p* ≤ 0.05; ** *p* ≤ 0.01; *** *p* ≤ 0.001; **** *p* ≤ 0.0001.

Considering the substantial involvement of the CDS in melanoma miRNA–mRNA interactions, we set out to compare mRNAs primarily targeted at the CDS with those targeted at the predicted and canonical 3′UTR. As most of the identified hybrids exhibited either non-predicted or non-canonical miRNA-mRNA interaction patterns, 22 targets of 4 miRNAs (let-7a, miR-100, miR-320a, and miR-34a) were selected for functional validations in the A375 melanoma cell line (Figure 6 and Figure S2), as well as in two additional pigmented melanoma cell lines, IGR37 and 501Mel (Figure S5). The aim was to determine potential differences in downregulation of miRNA targets by canonical versus non-canonical miRNA seed pairing and depending on whether the CDS or the 3′UTR was involved (Figure 6, Figure 7 and Figure S5). Some of the selected miRNAs have the same target genes (e.g., AXL), yet they target the corresponding mRNA at different regions (e.g., AXL is targeted at the 3′UTR by miR-34a, and at the CDS by miR-100 and let-7a) (Figure 6A). Along these lines, miR-34a targets AXL-3′UTR mostly via canonical seed pairing and strong supplemental pairing, while miR-100 and let-7a, which target the AXL-CDS tend to bind via non-canonical seed pairing involving strong supplementary binding (Figure 6B). Once again, this suggests that miRNAs interacting with the CDS and those targeting the 3′UTR appear to have different binding patterns with distinct functional outcomes. Overall, these genes (Figure 6) had high expression levels in melanoma patient data [39] (Figure S3) and negligible differences between normal tissues and primary or metastatic tumors (Figure S3A). Similarly, the expression levels of these genes were comparable in BRAF-, NRAS-, and NF1-mutant, as well as triple wild-type melanoma tumors (Figure S3B) indicating that the presence of mRNAs in qCLASH hybrids is not influenced by stage of disease or mutational background.

**Figure 8:**
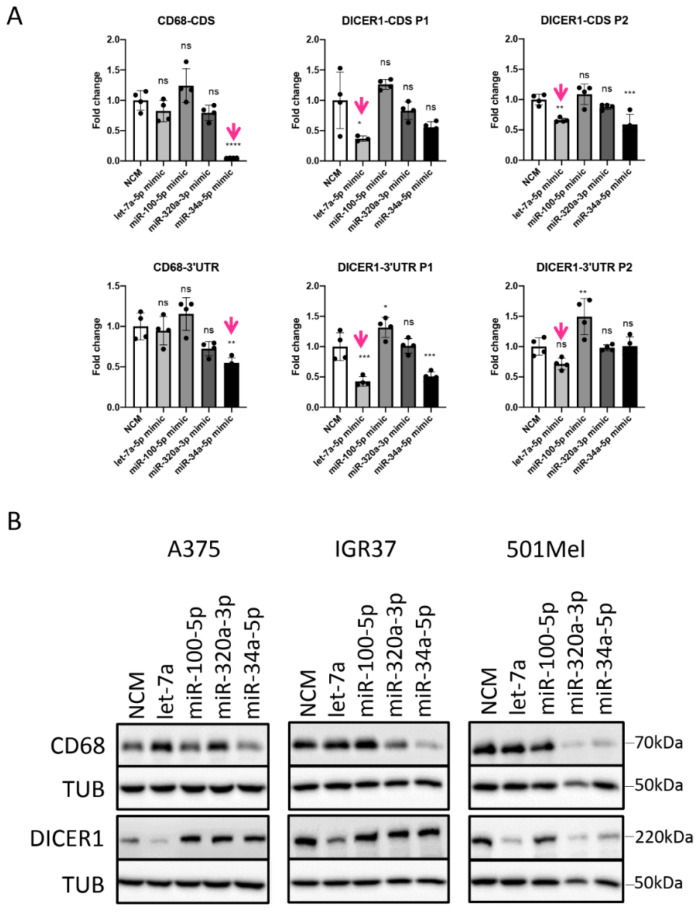
Experimental validation of miRNA–mRNA interactions. (**A**) Luciferase reporter vectors containing stretches of the target CDS or 3′UTR for selected miRNAs were transfected into A375 cells together with 50 nM miRNA mimic or NCM for 72 h. Illustrated are the mean ± standard deviation (SD) of at least three replicates. Statistical significance was determined by one-way ANOVA, followed by Dunnett’s multiple comparisons test with ns, not significant; * *p* ≤ 0.05; ** *p* ≤ 0.01; *** *p* ≤ 0.001; **** *p* ≤ 0.0001. P1 and P2 represent the parts of the cloned region. (**B**) Protein expression levels of selected miRNA targets upon 50 nM miRNA mimic or NCM treatment for 72 h in three different melanoma cell lines.

To validate potential functional effects, four miRNAs were selected based on their frequent occurrence in miRNA-mRNA hybrids (i.e., let-7a, and miR-320a), high expression in melanoma cells (i.e., let-7a, and miR-100), as well as their involvement in melanoma drug resistance (i.e., miR-34a, and miR-100 [39]). Transfection of mimics resulted in high intracellular levels of the respective miRNAs albeit to various degrees (Figure S4A). Among their respective interaction partners found in miRNA-mRNA hybrids, 22 mRNAs were selected for validation, in order to identify potential functional differences between canonical (i.e., 3′UTR) and non-predicted and non-canonical (i.e., binding patterns) (Figure 6). A third of the target genes were significantly downregulated (Figure 7A), two were upregulated (TUSC2, and MDM2) while the majority were not affected by the transfected mimics (Figure 7B). The downregulated genes mainly displayed MREs in the 3′UTR of the mRNA (Figure 6A) and the binding was accomplished via the canonical seed sequence (Figure 6B), most efficiently by miR-34a-5p. This is exemplified by AXL, which was not downregulated by miR-100 treatment (binding to CDS), while miR-34a mimics (binding to 3′UTR) reduced AXL mRNA amounts by 75% (Figure 7A). Although most of the herein tested CDS-mediated interactions did not result in reduced levels of target mRNAs (Figure 7B), some non-canonical interactions were clearly capable of down-regulating the target on mRNA and protein level, which we also confirmed by luciferase assays (e.g., let-7a-DICER1, and miR-34a-CD68) (Figure 7A and Figure 8).

### 3.5. miRNAs Regulating Important Pathways in Melanoma

**Figure 9:**
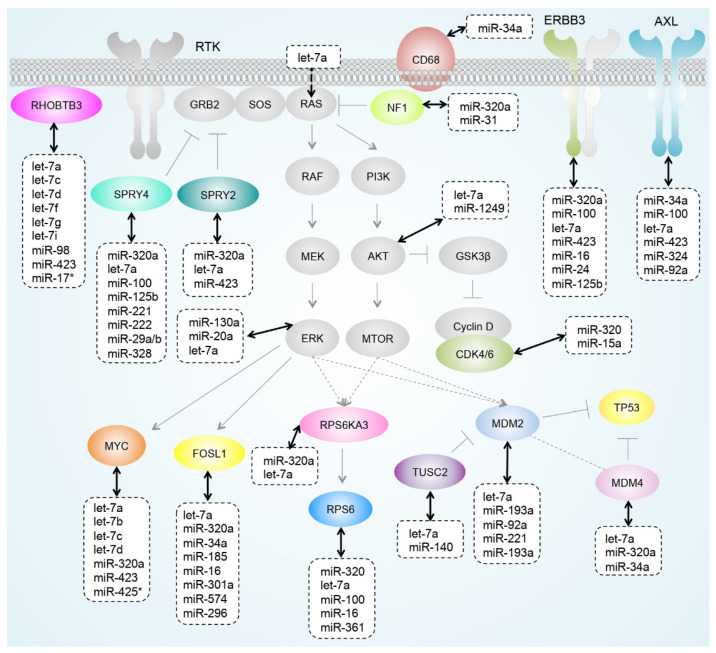
Overview of selected miRNA–mRNA interactions identified by qCLASH in melanoma cells. The graph summarizes herein and previously identified miRNA–target gene interactions, which are part of important signaling pathways in melanoma.

miRNAs have been shown to play crucial roles in melanoma growth and progression and can modulate pathways involved in drug resistance [13,42]. With qCLASH, we could identify predicted (miR-34a/AXL-3′UTR) and non-predicted miRNA–mRNA interactions (miR-34a/CD68-CDS) (Figure 9) as well as miRNA interactions with several receptor tyrosine kinases (RTKs) such as ERBB3 and AXL (Figure 7 and Figure 9), which have been shown to be upregulated in response to drug treatment and the subsequent development of drug resistance in melanoma cells and patients [43,44,45]. Additionally, several interactions between miRNAs and negative regulators of the MAPK pathway (NF1, SPRY2, and SPRY4) were found. As an example, SPRY4 mRNA levels were significantly reduced upon transfection of previously un-predicted miRNA mimics miR-100-5p, -34a-5p, and -320a-3p (Figure 7, Figure 8 and Figure 9). Interestingly, we observed a significant upregulation of the genes TUSC2 and MDM2 following miR-34a-5p mimic transfection while MDM4 was downregulated by the same miRNA, indirectly suggested by the presence of miR-34a-MDM4 in qCLASH hybrids (Figure 6). The upregulation of TUSC2 and MDM2 is likely an indirect effect of the miR-34a-mediated downregulation of MDM4. MDM2 and MDM4 are both regulators of the tumor suppressor protein p53 [46,47]. Inactivation of p53 in many cancers is generally brought about by mutations but can also result from increased levels of its inhibitors MDM2 and MDM4 [47], which dimerize to promote p53 degradation. TUSC2 (also known as FUS1), on the other hand, is an inhibitor of MDM2, blocking MDM2-associated proteolytic degradation of p53 [46,48]. The qCLASH data not only suggest that the expression of these players might be modulated by miRNAs, but also that the combined use of MDM2 and MDM4 antagonists would result in a more effective anti-tumor activity in p53 wild type cancers [46].

### 3.6. miRNA–lncRNA Interactions

**Figure 10:**
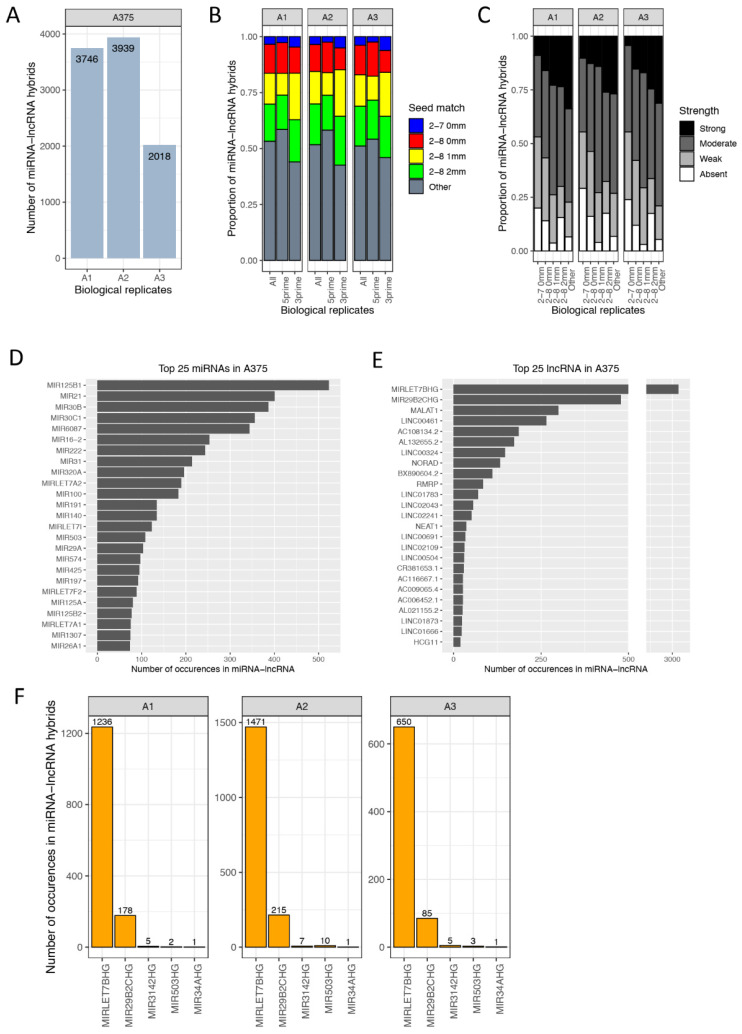
miRNA-lncRNA hybrids. (**A**) Number of miRNA-lncRNA hybrids in A375 replicates. (**B**) The type of seed pairing (5′ miRNA sequence) was divided and plotted as described in Figure 2E. The predicted and canonical seed sequence was displayed in blue (2–7 nt no mismatches) and red (2-nt no mismatches). Non-canonical seed pairing was displayed in yellow and green (one or two mismatches within the seed) or in grey for additional types of seed pairing (Other). (**C**) miRNA base-pairing via the 3′ portion of the miRNA (supplementary 3′ or non-seed pairing) as described in Figure 2F. The “strength” of binding outside the seed region at the 3′ end of the miRNA based on number of bound nt: >8 nt (strong), 5–8 nt (moderate), 1–4 nt (weak), and 0 nt (absent). Top 25 (**D**) miRNAs and (**E**) lncRNAs that occur in miRNA-lncRNA hybrids showing the number of hybrid sequences each RNA species occurs in. (**F**) Number of occurrences of pri-miRNA genes, which were labelled as lncRNAs in miRNA-lncRNA hybrids across three biological A375 replicates (A1, A2, A3).

As stated before, the qCLASH procedure generates hybrid data in an unbiased manner, especially with regard to the RNA species that are bound by the Ago protein. It has also previously been described that miRNAs do not only interact with protein-coding genes, but also with other small and long non-coding RNA molecules [18,20,49]. Consequently, next to miRNA-mRNA hybrids, we have discovered 2–4 K miRNA-lncRNA hybrids within the AGO complex, suggesting that several lncRNAs are interacting with miRNAs in melanoma cells (Figure 10A). While approximately 40% of miRNA–mRNA interactions occurred via canonical seed pairing (Figure 2E), only 25% of miRNA–lncRNA pairs involve canonical seed pairing, with negligible differences between 5′ and 3′ hybrids (Figure 10B), suggesting that distinct mechanisms govern miRNA-mediated regulation of lncRNAs and mRNAs. Furthermore, the importance of supplementary 3′ pairing of the miRNA is pronounced in miRNA-lncRNA hybrids, as illustrated by the increased strength in 3′ pairing in non-canonical seed interactions (Figure 10C). Among the miRNAs with the highest abundance in miRNA-lncRNA hybrids were miR-125, miR-21, let-7, and miR-16 (Figure 10D), similar to miRNA-mRNA hybrids (Figure 3B), however, with different ranks and abundance. The most frequently found lncRNAs was MIRLET7BHG, followed by MIR29B2CHG and MALAT1 (Figure 10E). As several miRNA genes were commonly detected in miRNA-lncRNA hybrids (e.g., MIRLET7BHG), we looked into additional potential primary miRNA transcripts that might interact with miRNAs themselves in an Ago-dependent manner. Five miRNA primary transcripts with very heterogeneous abundances and diverse miRNA binding patterns were identified within miRNA-lncRNA hybrids (Figure 10F). While miRNAs targeting MIR503HG use canonical seed binding in combination with a strong supplementary 3′ pairing, miRNAs targeting MIRLET7BHG show many different binding patterns with a preference for non-canonical binding. Most of the miRNA primary transcripts are interacting with miRNAs other than their mature miRNA (Figure S6). MIRLET7BHG, however, is targeted by many miRNAs among which are several members of the let-7 family (e.g., MIRLET7A1, MIRLET7C, and MIRLET7F2), indicating a potential auto-regulation.

On the whole, our data suggest that miRNA–target interactions go beyond the canonical miRNA-induced mRNA downregulation, also encompassing non-mRNA miRNA targets (e.g., lncRNA) as well as the highly abundant non-canonical miRNA–target interactions, some of which are functional in down-regulating their binding partner.

## 4. Discussion

Since the discovery of miRNAs in 1993 [50], extensive progress has been made in understanding their biogenesis, expression patterns, and function, as well as their role in fundamental physiological and pathological processes [6,51,52]. A recent comprehensive meta-analysis [2] of 28,866 small RNAseq data sets, has estimated the true number of miRNAs to be 2300 in humans, which are organized in 90 broadly conserved miRNA families [1,53]. Given that over 45,000 conserved canonical miRNA binding sites were found in 3′UTR of mRNAs [54] and that each miRNA can interact with multiple targets, the complexity of miRNA-mediated post-transcriptional regulation of gene expression becomes evident. Recent advancements in ribonomics technologies, such as CLIP-based techniques (HITS-CLIP, CLEAR-CLIP, PAR-CLIP, CLASH, and qCLASH) allow for identification of miRNA binding sites and have revealed a considerable number of non-canonical interactions between miRNAs and their targets, thus broadening the current understanding of the miRNA targetome [17,18,55,56,57,58,59]. Nevertheless, the extent to which non-canonical interactions, i.e., interactions diverting from the seed sequence pairing with partial or extensive complementarity in the 3′UTR of mRNAs, are contributing towards measurable and functional down-regulation of coding or non-coding RNA targets is not fully understood and currently under investigation. First evidence on the still scarce datasets suggests that only a subset of such non-canonical interactions may have functional impact [1,14,17,60].

In this study, we investigated non-predicted and non-canonical miRNA–target gene interactions in melanoma with the aim to analyze binding characteristics of miRNAs in cancer cells. To our knowledge, this is the first application of qCLASH to cancer cells resulting in the identification of 50–95 k miRNA-mRNA hybrids, many of which displayed non-canonical interaction characteristics that go beyond the miRNA seed sequence and the mRNA-3′UTR. Interestingly, there was a clear bias towards miRNA interaction targeting the CDS of mRNAs with 70% of miRNA-binding sites located outside of the 3′UTR. Although it has recently been suggested that most non-canonical miRNA–target interactions might simply reflect a low-affinity binding of miRNAs while moving along the RNA sequence and before arriving at higher affinity 3′UTR sites [1], some non-canonical interactions have been shown to have measurable effects on their targets [34,35]. Furthermore, the overall number of non-canonical sites in non-coding RNAs, pseudogenes, circular RNAs, CDS of mRNAs, etc. surely contributes to sequestration of miRNAs, which might result in stabilized higher expression of the canonical 3′ UTR targets. It remains to be shown which and how many non-canonical interactions are functional and whether this varies with cellular backgrounds and cell states and whether functional non-canonical encounters can eventually be predicted [61]. Generally, downregulated genes were more likely to have perfect seed matching in the 3′UTR, which reflects the current understanding of how miRNAs exert their function [1,7,62]. To investigate putative functional effects, we selected 4 miRNAs and 22 corresponding target genes for validation. Out of the selected genes, a third were significantly downregulated, whereas the majority of target gene expression levels were not affected by miRNA mimic treatment. We observed distinct characteristics between genes, which were downregulated or not upon miRNA transfection. Genes that were targeted at the 3′UTR were more likely to display reduced expression levels (e.g., miR-34a-5p and AXL-3′UTR), compared to those targeted at the CDS (e.g., miR-100-5p and AXL-CDS), with few exceptions (e.g., let-7a-5p and DICER1-CDS, miR-34a-5p and CD68-CDS). Of note, the effects of some miRNAs can only be demonstrated on protein level rather than mRNA level as they act on translation and not mRNA stability. In addition, miRNAs with canonical seed pairing were more likely to downregulate the putative target. Many non-canonical binding patterns were observed across all biological replicates and in three additional cell lines with distinct phenotypic features, suggesting that such interactions do not occur randomly, but might have a function beyond repression, such as “sponging” miRNAs or leading to miRNA degradation via target RNA-directed miRNA degradation (TDMD) [35,63,64,65]. Nevertheless, we could show that miRNAs targeting the CDS can directly lead to the reduction of gene expression levels as exemplified by miR-34a targeting the CDS of CD68. Interestingly, a recent study discovered a novel class of functional MREs in the CDS of the p53-activating kinase (DAPK3), which appear to require extensive 3′ base pairing with minimal 5′ seed pairing [34]. Additionally, these miRNAs caused gene silencing in an Ago-dependent and GW182-independent manner, thus not having an impact on mRNA stability but rather a global effect on translation as reflected by ribosome stalling [34]. Despite the previous observation that CDS-associated MREs are largely non-functional as compared to the 3′UTR [59], Zhang et al. suggest that miRNAs targeting the CDS use mechanisms that differ from those that target the 3′UTR; therefore, the mechanism of action and functional consequences of a miRNA depend on the location of its target site [34,35,66].

Moreover, we have identified a modest number of miRNAs interacting with long non-coding RNAs (lncRNAs) in our qCLASH data set in melanoma. LncRNAs are known to be involved in the modulation of cancer metastasis, metabolism, and immunity [67,68]. Of note, a direct interaction between miRNAs and lncRNAs that serve as substrates or miRNA precursors for the miRNA biogenesis pathway has previously been demonstrated [69,70]. The Argonaute protein ortholog in C. elegans, ALG-1, was shown to bind to a specific region at the 3′ end of let-7a primary transcripts (pri-let-7a), which is mediated by the mature let-7a miRNA through conserved complementarity in its own primary transcript. Interestingly, the interaction between ALG-1 and pri-let-7a was found in nuclear fractions, which was also observed for AGO in human cells. This once again demonstrates that in addition to protein-coding mRNAs, miRNAs can target other non-coding RNAs in different species [71]. In this context, several studies have demonstrated that AGO-bound miRNAs can be shuttled into the nucleus in order to exert their function, which is in line with the different types of miRNA targets that have been observed in qCLASH hybrids [3,72,73]. Hence, qCLASH allows for capturing AGO-bound miRNAs in the cytoplasm as well as in the nucleus. Furthermore, most of the identified miRNA–lncRNA interactions followed non-canonical binding characteristics, as for instance, miRNAs targeting MIR503HG use canonical seed binding in combination with a strong supplementary 3′ pairing suggesting that supplemental 3′ pairing might compensate for imperfect seed pairing in both mRNA and lncRNA targets. Despite recent evidence and the common belief that supplemental 3′ pairing is a poorly conserved rarity with minor influence, which in some cases can compensate imperfect seed pairing [1,60], a number of studies highlight its importance in determining target specificity [56,59,74]. A recent study has emphasized the importance of supplemental 3′ pairing, and suggests that optimal supplemental pairing can increase target affinity more than 20-fold [74]. Subsequently, the supplemental 3′ pairing does not only provide a mechanism for extended miRNA targeting, but could also guide target specificity of 3′ isomiRs and miRNA family members [56,74], indicating that these supplementary interactions contribute more to target recognition than is appreciated so far [75,76].

In cancer cells miRNAs are often deregulated resulting in distinct disease-specific expression profiles [77,78], which have spurred the development of preclinical miRNA therapeutics to modulate their expression levels [79]. The identification of effective therapeutic miRNA candidates, specific and effective delivery methods, and toxicity are among some of the remaining obstacles in miRNA therapeutics [79]. An essential pre-requisite for developing miRNA-based drugs, is a deeper understanding of the functional impact miRNAs can have on transcriptional networks, which fine-tune gene expression and this can only be achieved with more accurate data on their cell- and disease-specific targetomes. Modified CLIP-based techniques such as qCLASH can contribute such unbiased and large-scale interaction data of cells of interest. Albeit a powerful method, CLASH data would benefit from technical improvements, especially on the intermolecular RNA-RNA ligation step, which we are currently working on. In future, CLASH-like data will likely be incorporated into standard target gene prediction algorithms, as they capture real RNA–RNA interactions in an impartial manner.

## 5. Conclusions

As miRNAs are involved in oncogenic processes by regulating many oncogenes and tumor suppressors, the reliable identification of miRNA target genes and their mechanisms of action remain a crucial but unsolved task. To our knowledge, we describe here for the first time, the unbiased investigation of the miRNA targetome in cancer cells by direct RNA-RNA ligation using a CLASH-based technique. We show that (i) the majority of miRNA-target interaction deviate from the canonical and predicted miRNA targetome, (ii) in over 50% of cases miRNAs have a clear bias towards to CDS of the target mRNA, (iii) up to 60% of miRNA target interaction occur via non-canonical seed pairing, (iv) there is a strong contribution of the 3′ miRNA sequence in the binding to target RNA, (v) miRNAs targeting the CDS can directly lead to the reduction of gene expression, while (vi) the majority of non-canonical miRNA interactions seems to have roles beyond target gene suppression, (vii) qCLASH allowed to identify non-mRNA targets of miRNAs (e.g., lncRNA), and (viii) the interaction between miRNAs and lncRNAs mainly occurs via non-canonical binding characteristics.

## Data Availability

The datasets generated during the current study have been submitted to the ArrayExpress repository under the accession number E-MTAB-8591 (http://www.ebi.ac.uk/arrayexpress/experiments/E-MTAB-8591).

## Supplementary Materials

The following are available online at https://www.mdpi.com/2072-6694/13/5/1096/s1, Figure S1: miRNA-mRNA hybrid data of additional melanoma cells. Figure S2: Overview of binding characteristics of miRNA-mRNA hybrids. Figure S3: Expression of selected genes in melanoma patient TCGA data. Figure S4: Expression of selected target mRNAs following miRNA mimic transfection. Figure S5: Expression of selected target mRNAs following transfection with 4 different miRNA mimics in IGR37 (A) and 501Mel (B). Figure S6: Detailed overview of pri-miRNA–mRNA interactions. Table S1: qCLASH data: facts and figures. Table S2: Detailed overview of miRNA-mRNA hybrids as well as binding characteristics. Table S3: Numerical data as illustrated in Figure 5.

## Abbreviations

qCLASH: cross-linking ligation and sequencing of hybrids; RISC: RNA-induced silencing complex; 3′UTR: 3′ untranslated region; MRE: miRNA recognition element; lncRNA: long non-coding RNAs; CDS: coding DNA sequence; AGO: Argonaute; RNP: RNA-protein complex; NCM: negative control mimic; nt: nucleotide; mm: mismatch.
