# Supplementary material for: Cross-Linking Ligation and Sequencing of Hybrids (qCLASH) Reveals an Unpredicted miRNA Targetome in Melanoma Cells
Source: Cancers (Basel). 2021 Mar 4;13(5):1096. doi: 10.3390/cancers13051096 (PMC7961530; doi:10.3390/cancers13051096)
Supplement: Supplementary file 1 [file cancers-13-01096-s001.zip › supp+wb/revised supp/Kozar_et_al_supp_figures_2021_02_18.pdf]

Cross-linking ligation and sequencing of hybrids (qCLASH) reveals an unpredicted miRNA targetome in melanoma cells

Ines Kozar[1], Demetra Philippidou[1], Christiane Margue[1], Lauren A. Gay[2], Rolf Renne[2], Stephanie Kreis[1]

[1] Department of Life Sciences and Medicine, University of Luxembourg, 6, avenue du Swing, L-4367 Belvaux, Luxembourg
[2] Department of Molecular Genetics and Microbiology, University of Florida, 1200 Newell Drive, Gainesville, FL 32610, USA

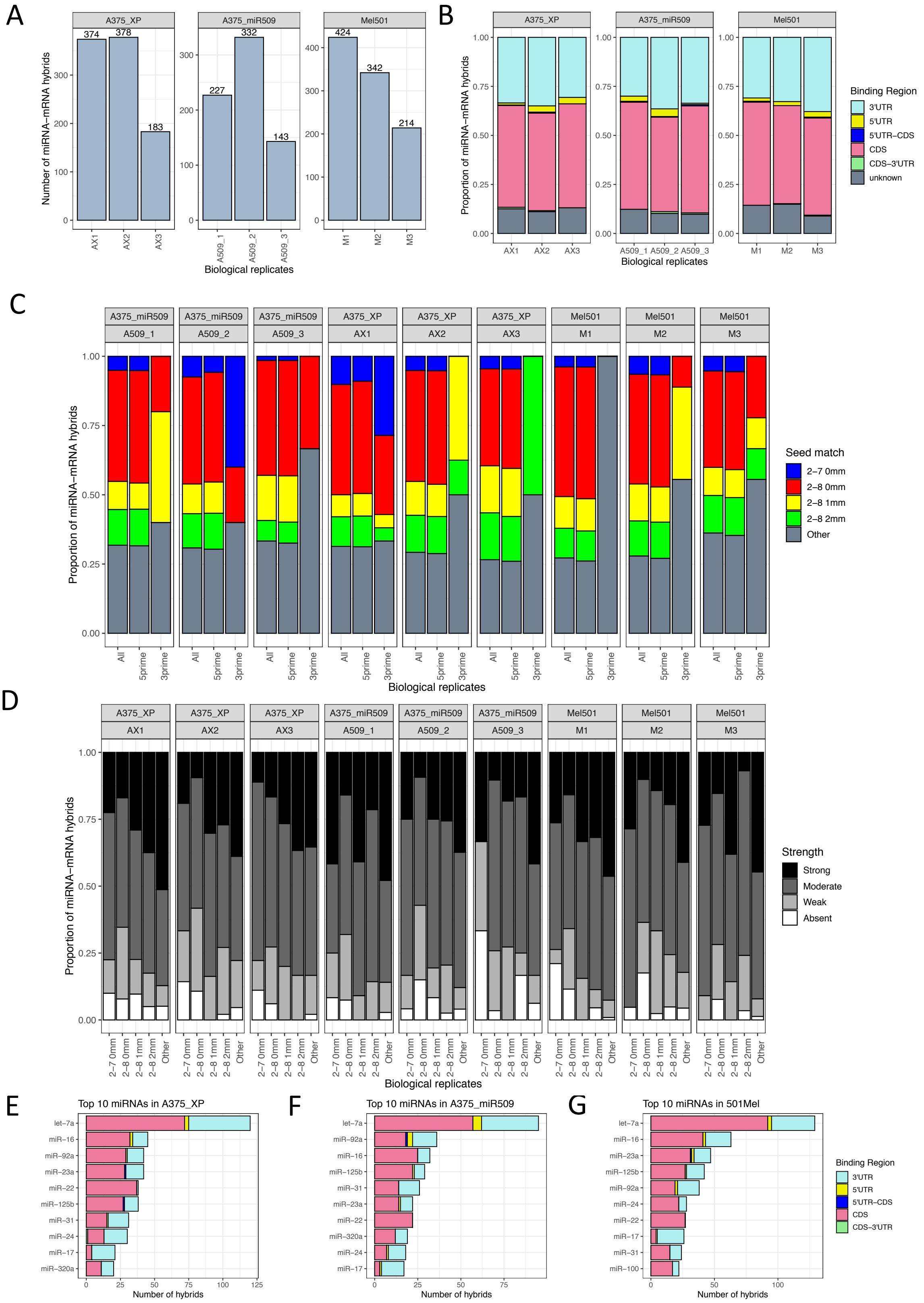

Figure S1: (Caption on next page.)

Figure S1: (Previous page.) **miRNA-mRNA hybrid data of additional melanoma cells.** (A) Number of miRNA-mRNA hybrids identified in the three biological replicates of additional BRAF-mutant melanoma cell lines. A375_XP (Vemurafenib-resistant) and A375_miR509 (over-expressing miR-509) are cells derived from the parental cell line A375. 501Mel is an independent highly pigmented BRAF-mutant melanoma cell line. (B) Proportion of miRNA-mRNA hybrids across three biological replicates of the different melanoma cells with miRNA binding sites mapped to the mRNA sequence and the number of identified hybrid sequences in each replicate. (C) The type of seed pairing (5' miRNA sequence) was divided into classes consisting of nt 2-7 at 5' end of miRNA with zero mismatches (2-7 0mm), nt 2-8 with zero mismatches (2-8 0mm), one mismatch (2-8 1mm), or two mismatches (2-8 2mm), and the remaining binding modes were classified as other. The proportion of miRNA-lncRNA hybrids across the three biological replicates and for all hybrids, 5' and 3' hybrids was plotted for the different seed categories. (D) miRNA base-pairing via the 3' portion of the miRNA (supplementary 3' or non-seed pairing). The strength of 3' sequence binding was classified as strong (>8 bound nt), moderate (5-8 bound nt), weak (1-4 bound nt), and absent (zero bound nt) and was plotted for each seed sequence type across the biological replicates. Top 10 miRNAs that occur in miRNA-mRNA hybrids showing the number of hybrids detected and the corresponding binding region on the mRNA in (E) A375_XP cells, (F) A375_miR509 cells (stably transfected with miR509), and (G) 501Mel cells.

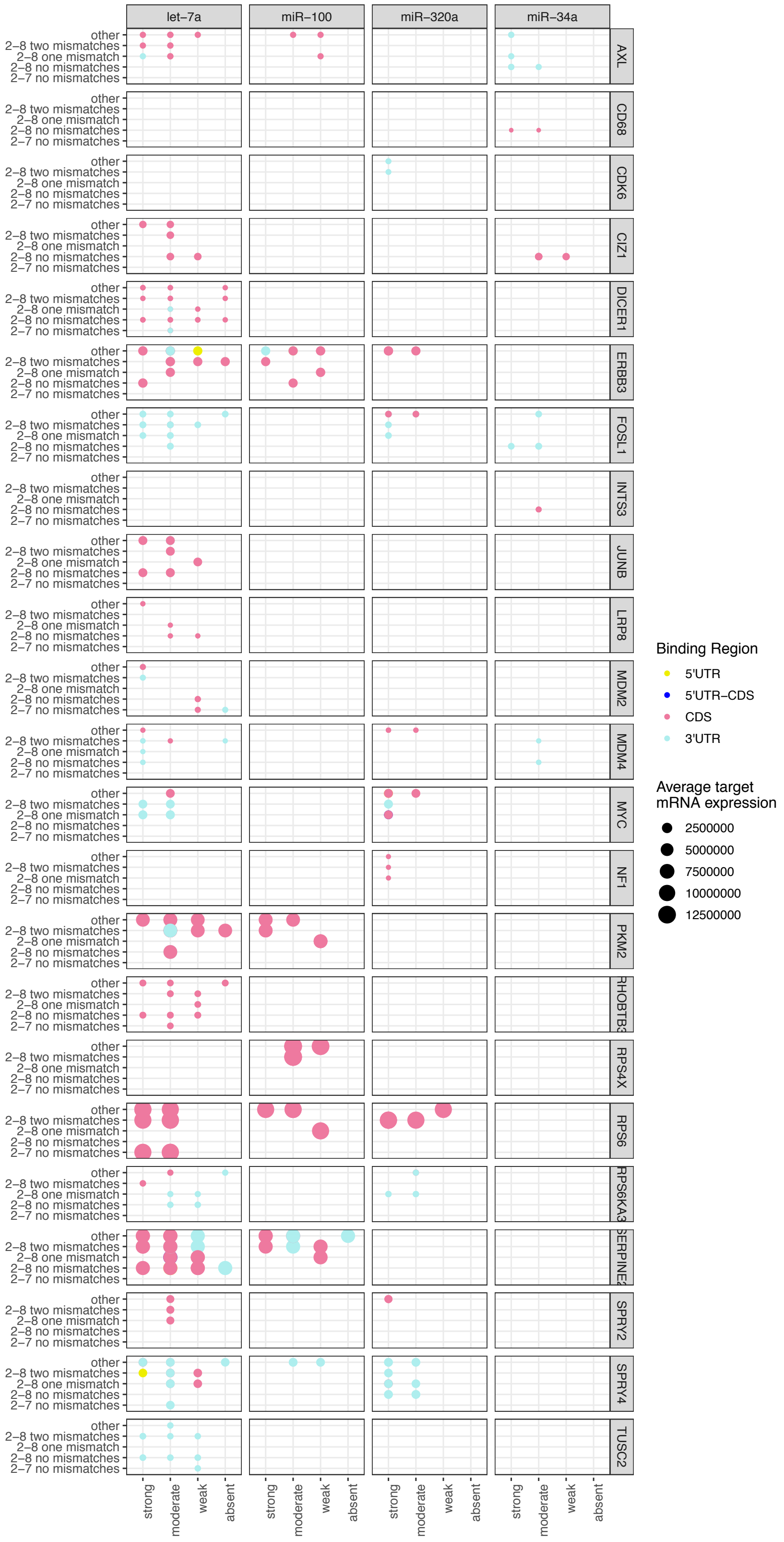

Figure S2: (Caption on next page.)

Figure S2: (Previous page.) **Overview of binding characteristics of miRNA-mRNA hybrids.** Selected miRNA-mRNAs with the corresponding mRNA expression levels in melanoma patient data (TCGA2015). The binding characteristics (seed match, supplemental 3' pairing or strength, and binding region on mRNA) are illustrated as well as the average target mRNA expression levels.

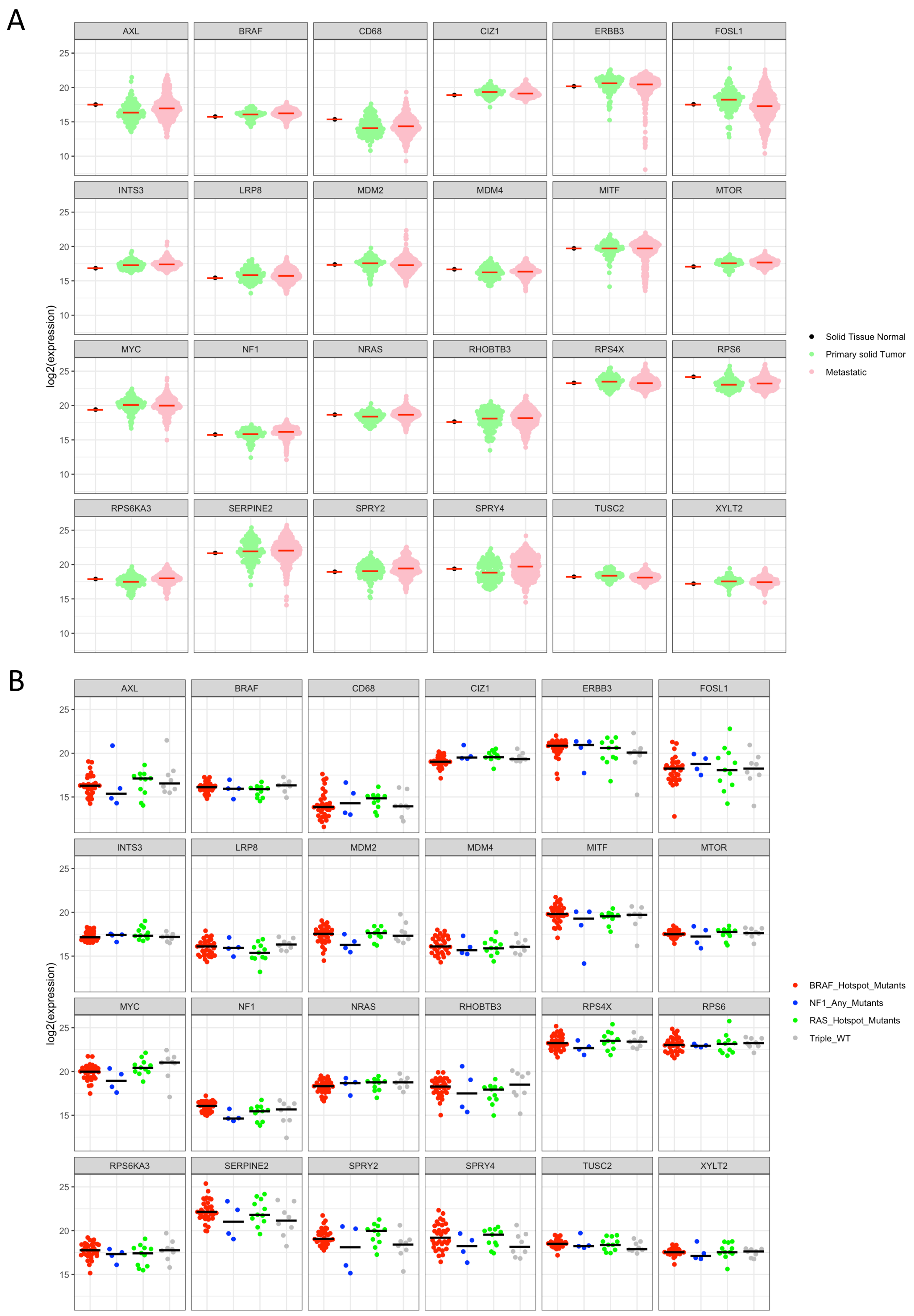

Figure S3: (Caption on next page.)

Figure S3: (Previous page.) **Expression of selected genes in melanoma patient TCGA data.** Expression of selected genes found in hybrids in TCGA melanoma patient data according to (A) tissue origin (normal solid tissue, primary solid tumor, metastatic tumor) and (B) mutational subtype (BRAF-mutant, NF1-mutatnt, RAS-mutant, or triple wild type melanoma tumours).

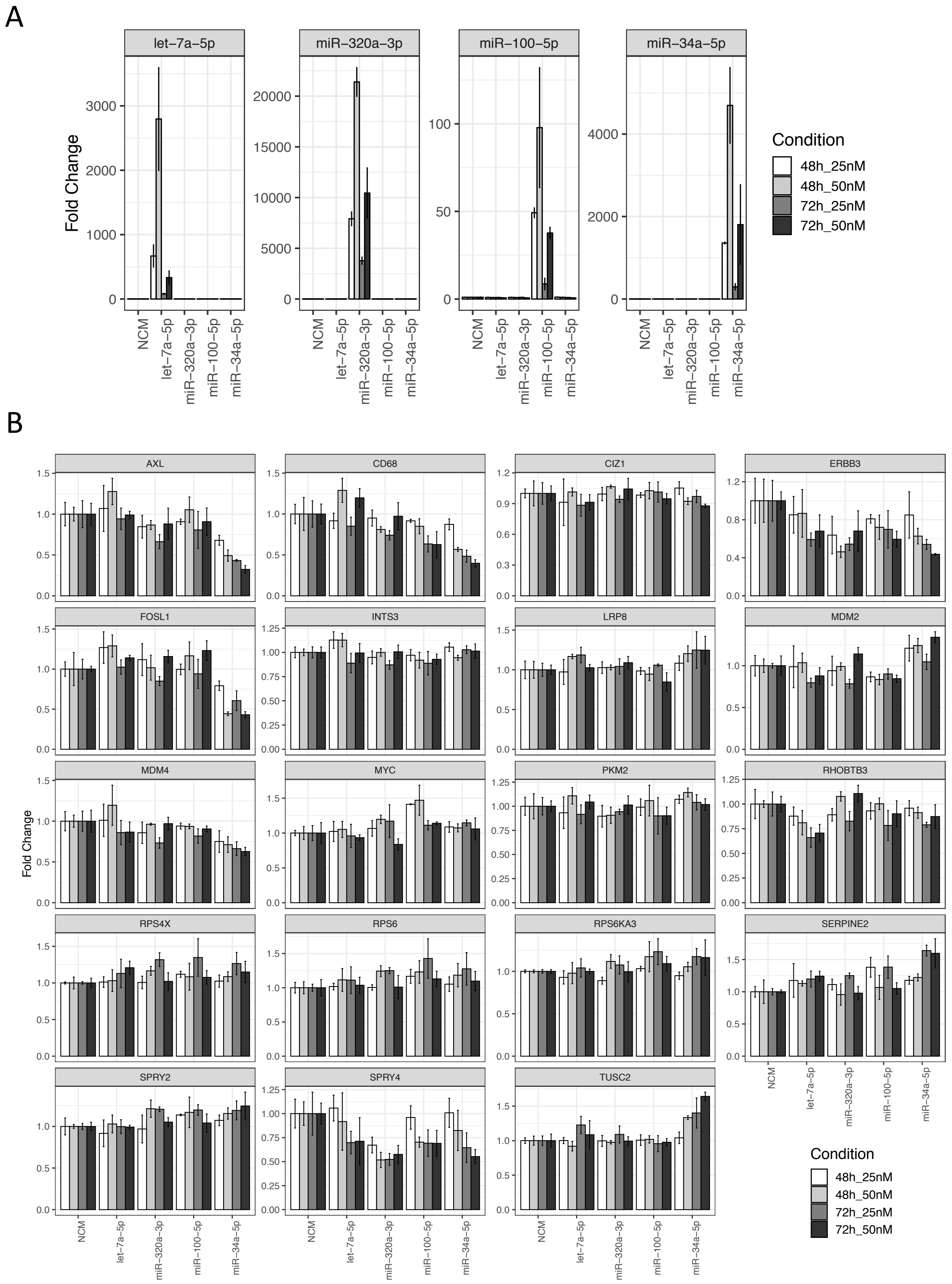

Figure S4: (Caption on next page.)

Figure S4: (Previous page.) **Expression of selected target mRNAs following miRNA mimic transfection.** (A) The expression of four selected miRNAs in A375 cells transfected with 25nM and 50nM miRNA mimic for 48h and 72h compared to cells treated with negative control mimic (NCM). Represented are the mean and standard deviation of three technical replicates (n=1). (B) The expression fold change of selected target mRNA upon miRNA mimic transfection was normalised to the corresponding negative control mimic (NCM) in each condition (25nM and 50nM mimic transfection for 48h and 72h). Represented are the mean and standard deviation of three technical replicates.

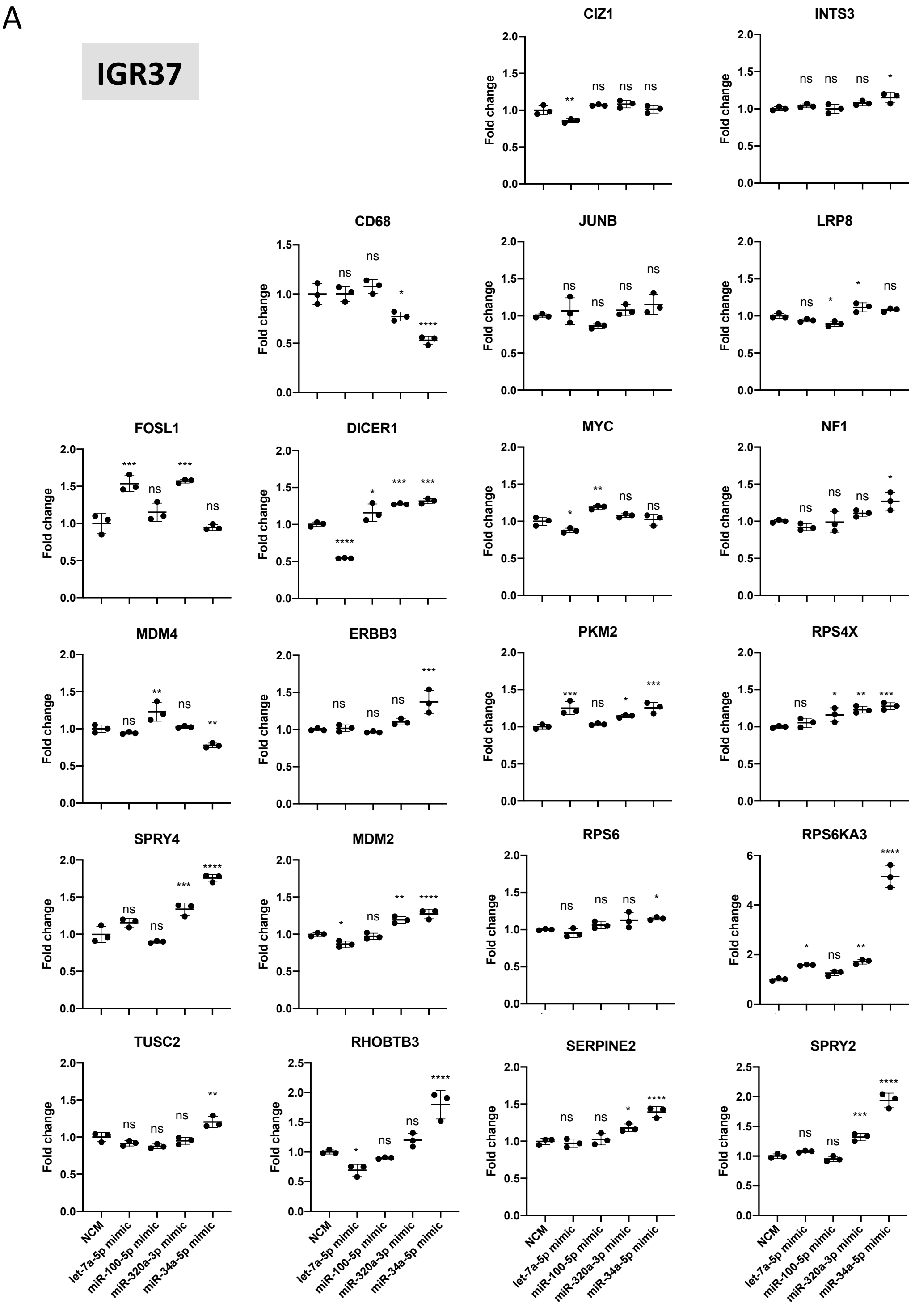

Figure S5: (Caption on next page.)

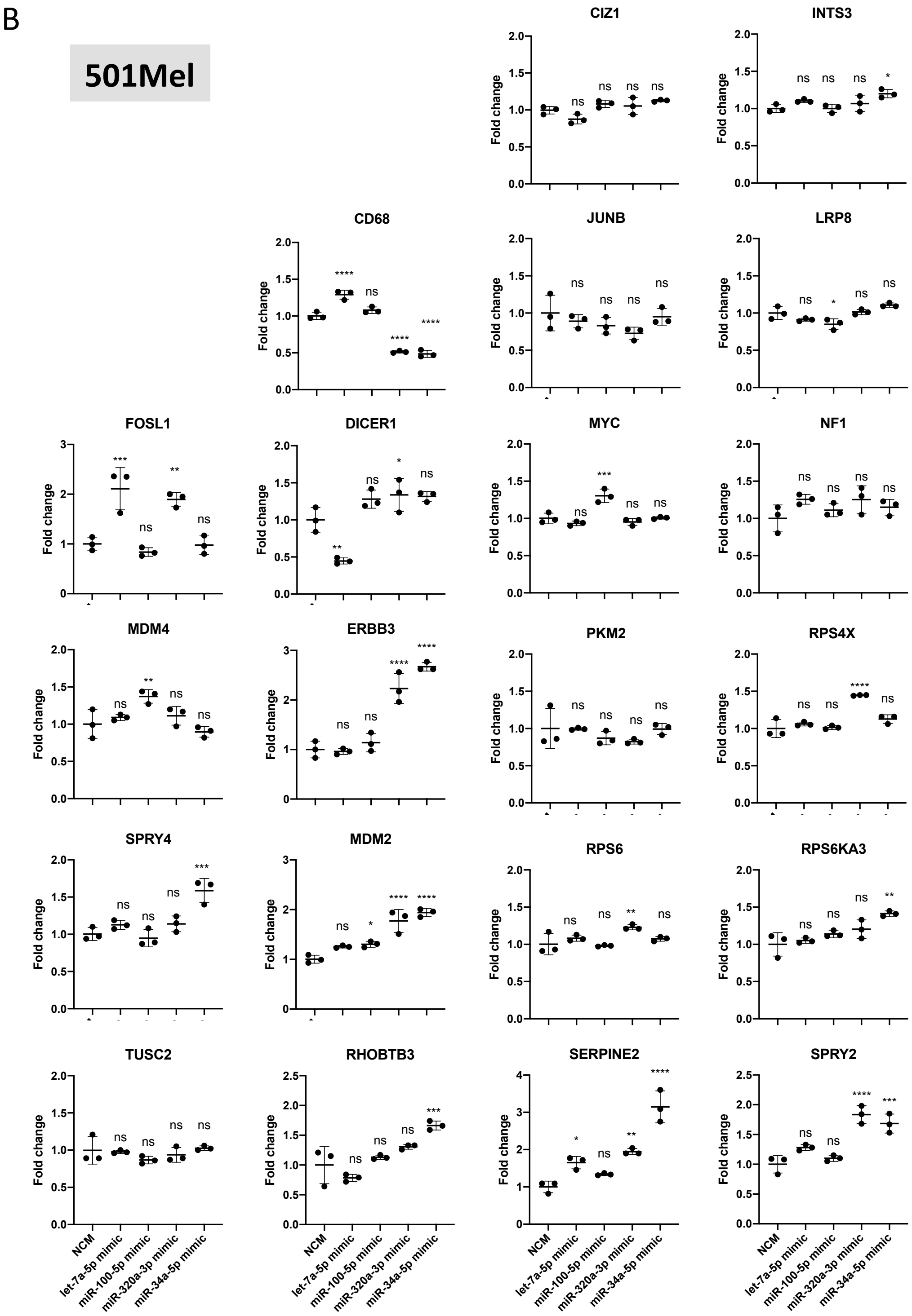

Figure S5: (Caption on next page.)

Figure S5: (Previous page.) **Expression of selected target mRNAs following transfection with 4 different miRNA mimics** in IGR37 (A) and 501Mel (B). The expression levels were normalized to the corresponding negative control mimic (NCM). Illustrated are the mRNA expression levels of 21 genes upon 50nM mimic treatment for 72h. Statistical significance was determined by one-way ANOVA, followed by Dunnett's multiple comparisons test with ns, not significant; *, P $\leq$ 0.05; **, P $\leq$ 0.01; ***, P $\leq$ 0.001; ****, P $\leq$ 0.0001.

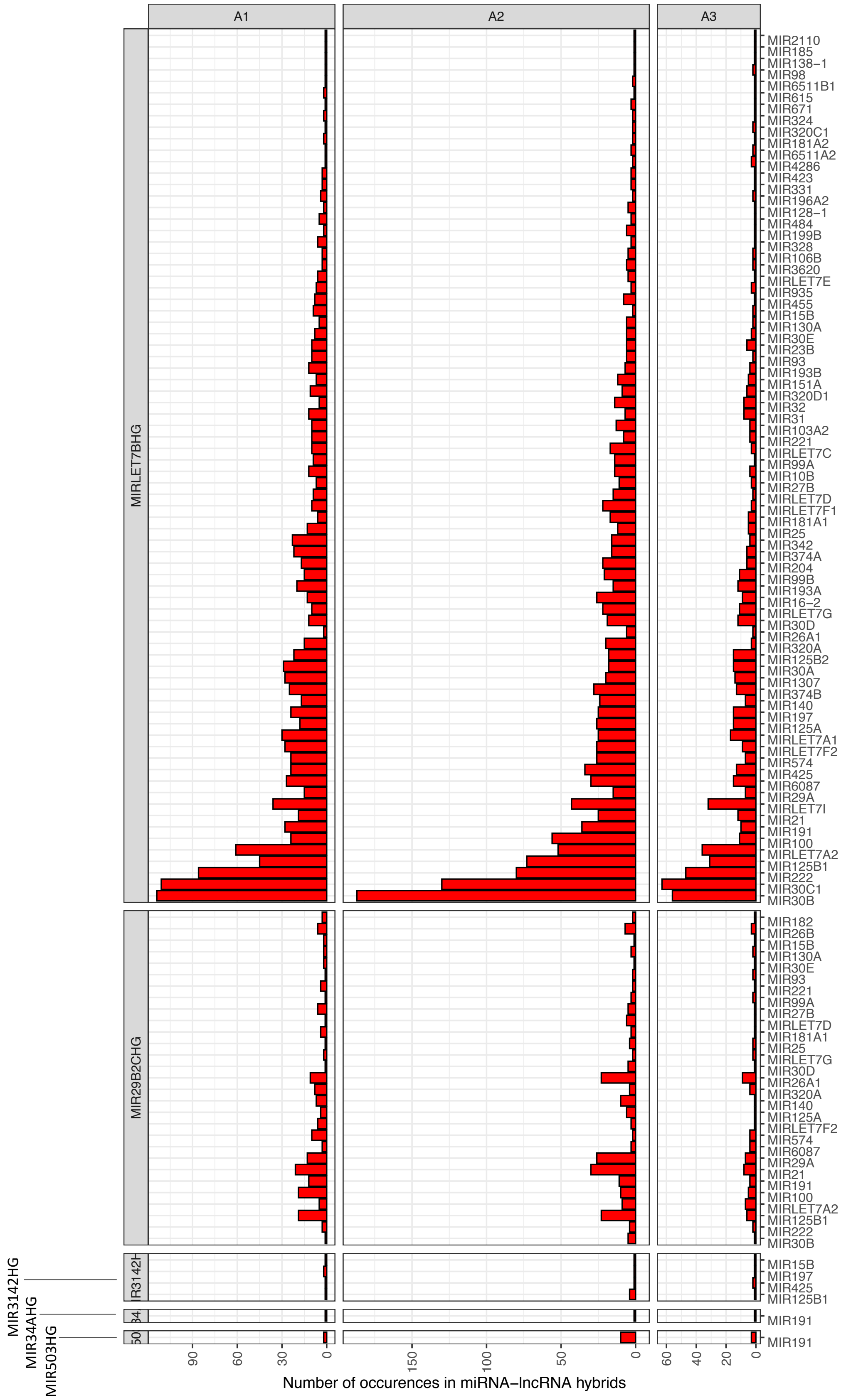

Figure S6: (Caption on next page.)

Figure S6: (Previous page.) **Detailed overview of pri-miRNA-miRNA interactions.** Detailed overview of miRNAs that have been found to interact with the most frequent pri-miRNAs (lncRNAs: MIRLET7BHG, MIR29B2CHG, MIR3142HG, MIR503HG, MIR34AHG). The number of occurrences of the corresponding miRNAs in miRNA-lncRNA hybrids is illustrated across three biological replicates and pri-miRNA genes.
