# Supplementary material for: Cross-Linking Ligation and Sequencing of Hybrids (qCLASH) Reveals an Unpredicted miRNA Targetome in Melanoma Cells
Source: Cancers (Basel). 2021 Mar 4;13(5):1096. doi: 10.3390/cancers13051096 (PMC7961530; doi:10.3390/cancers13051096)
Supplement: Supplementary file 1 [file cancers-13-01096-s001.zip › supp+wb/WB.pdf]

Cross-linking ligation and sequencing of hybrids (qCLASH) reveals an unpredicted miRNA targetome in melanoma cells

Kozar *et al.*,

Supplements

Original western blot images corresponding to Figure 8B

Parts of the blot that are shown in the manuscript are highlighted by a red box

The original and non-annotated images are in the attached zipped folder

Blot 1

A375

CD68

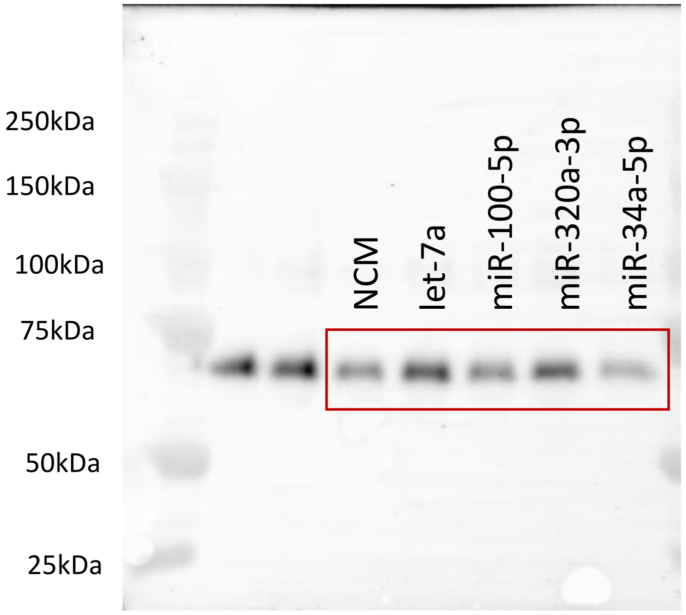

TUB

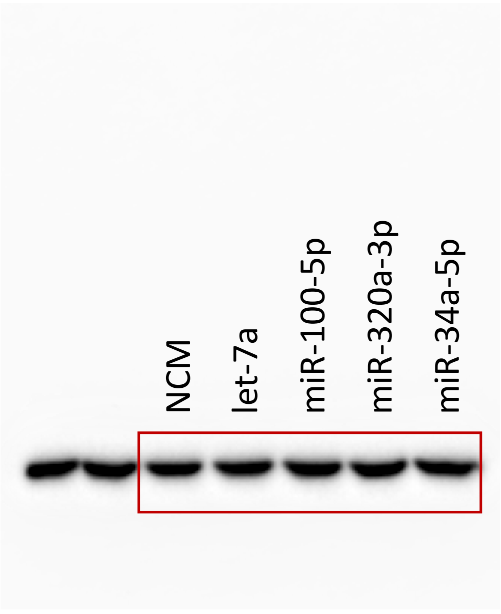

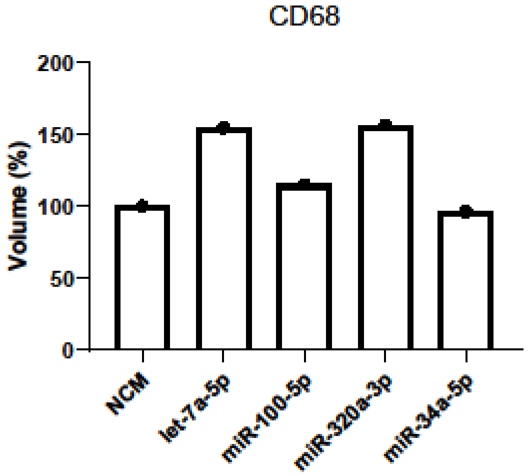

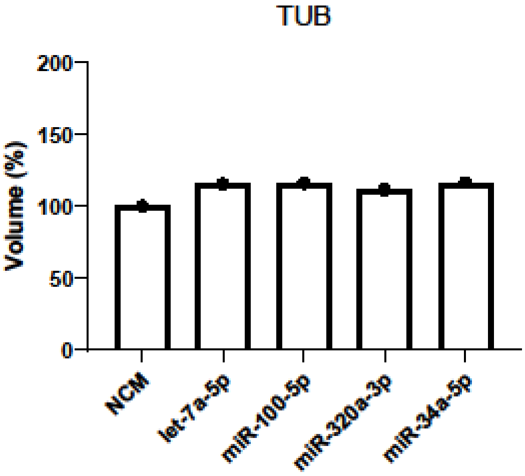

Protein bands have been quantified using the FusionCapt Advance software. The band intensity is expressed as the volume (%) relative to the intensity of the negative control mimic (NCM).

Blot 2

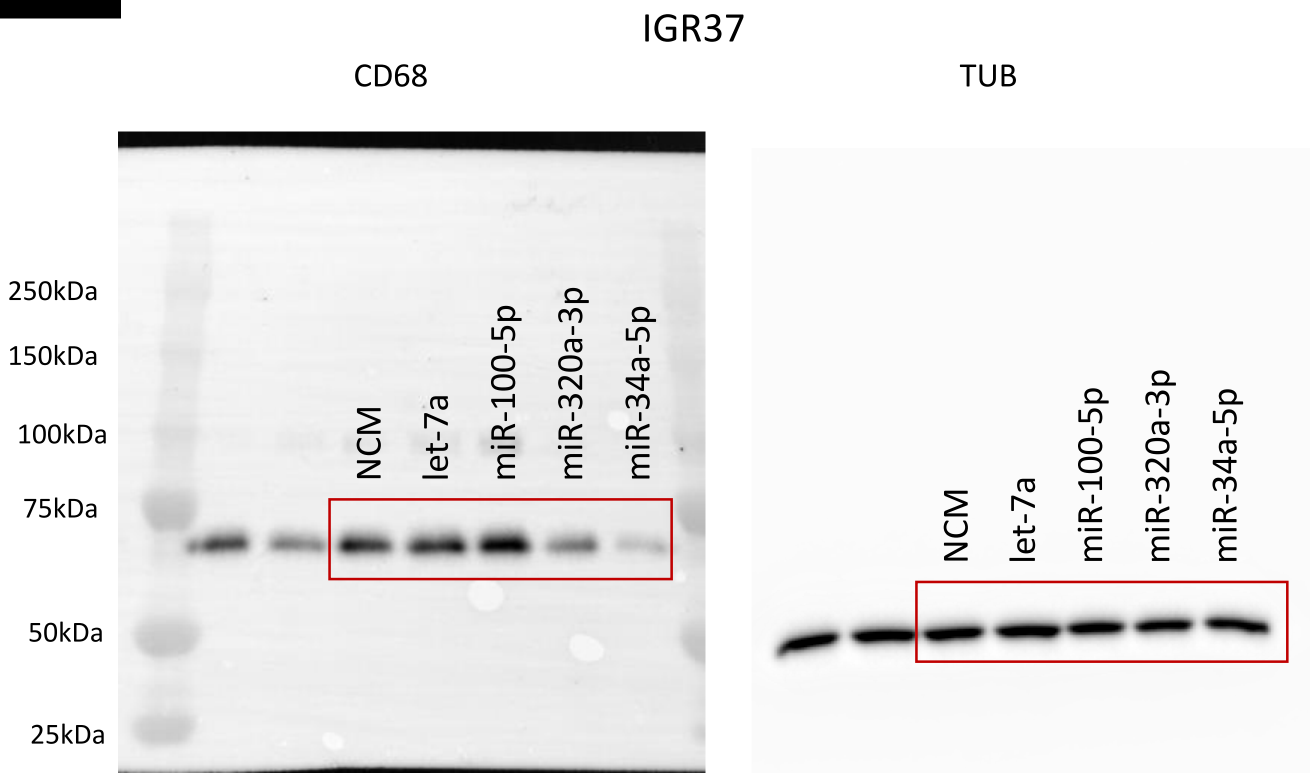

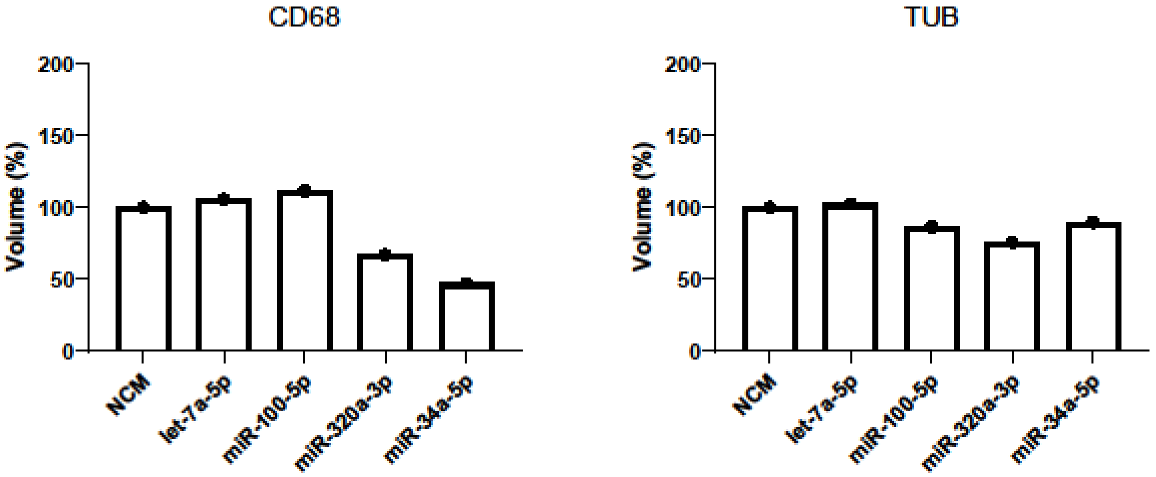

Protein bands have been quantified using the FusionCapt Advance software. The band intensity is expressed as the volume (%) relative to the intensity of the negative control mimic (NCM).

Blot 3

501Mel

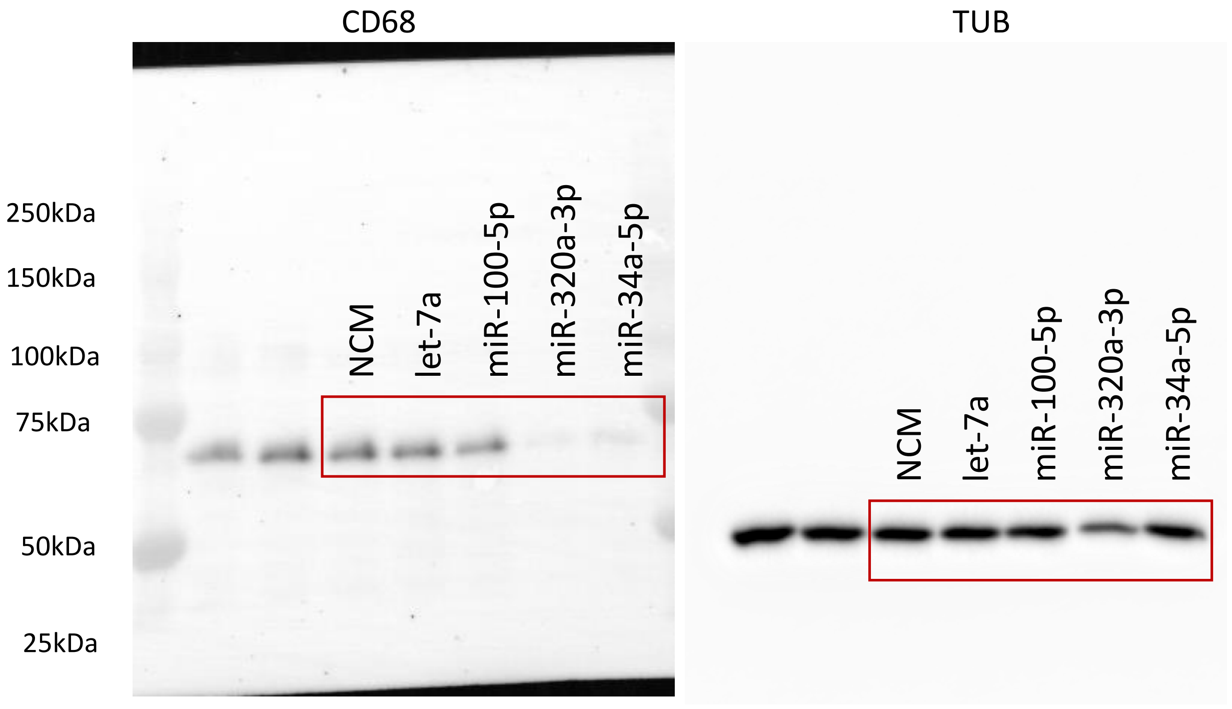

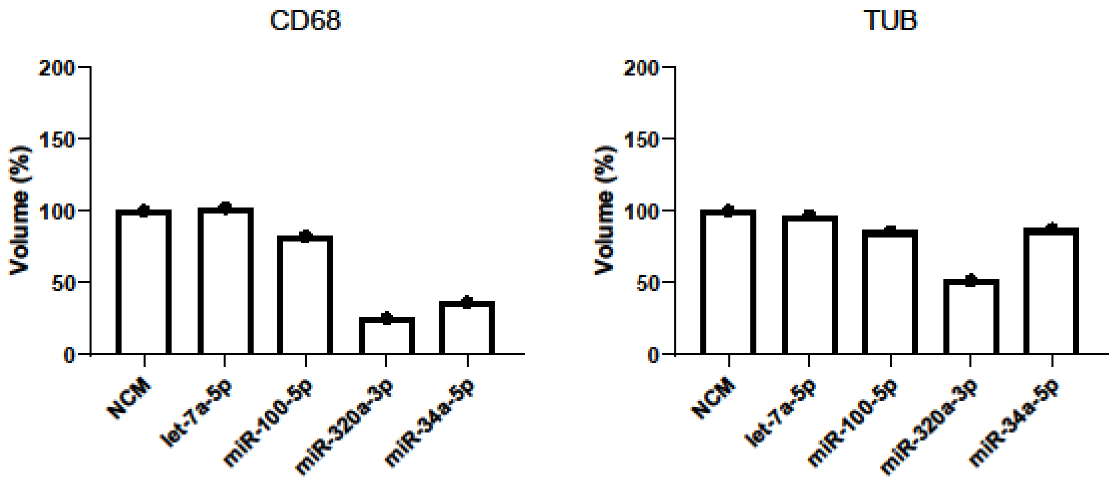

Protein bands have been quantified using the FusionCapt Advance software. The band intensity is expressed as the volume (%) relative to the intensity of the negative control mimic (NCM).

Blot 4

A375

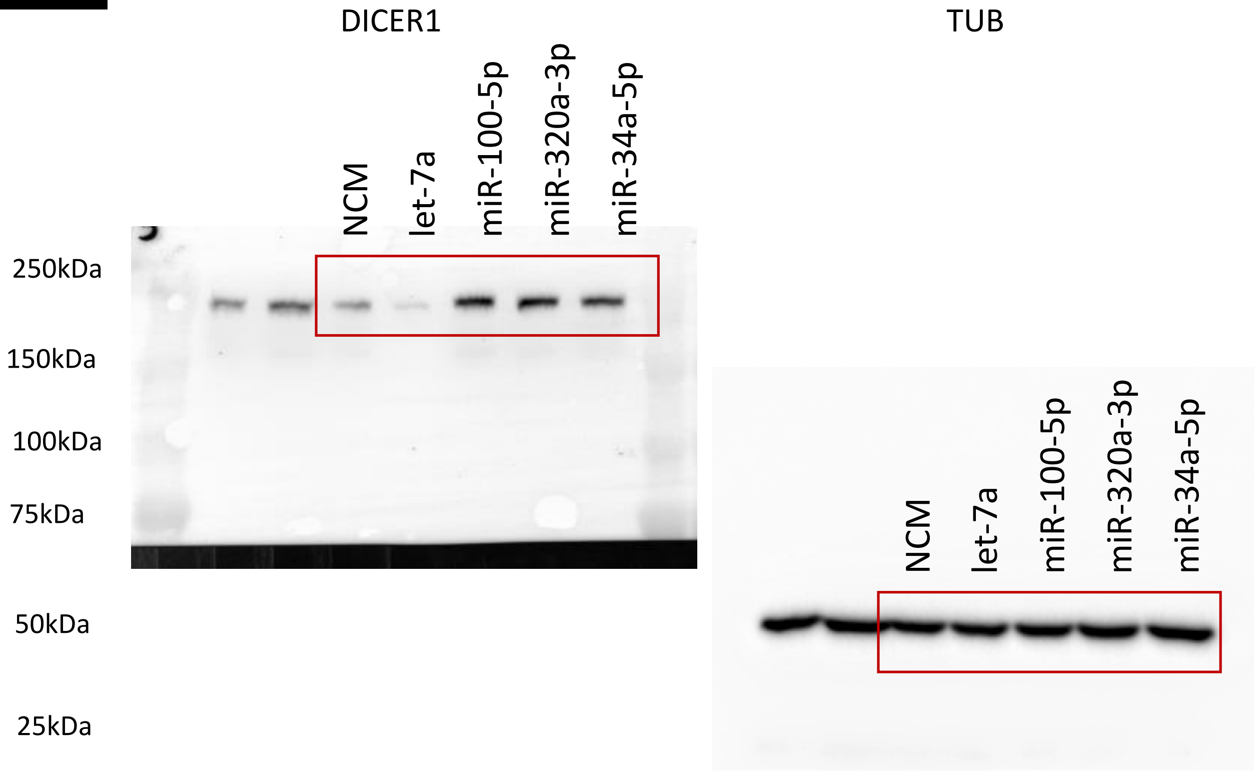

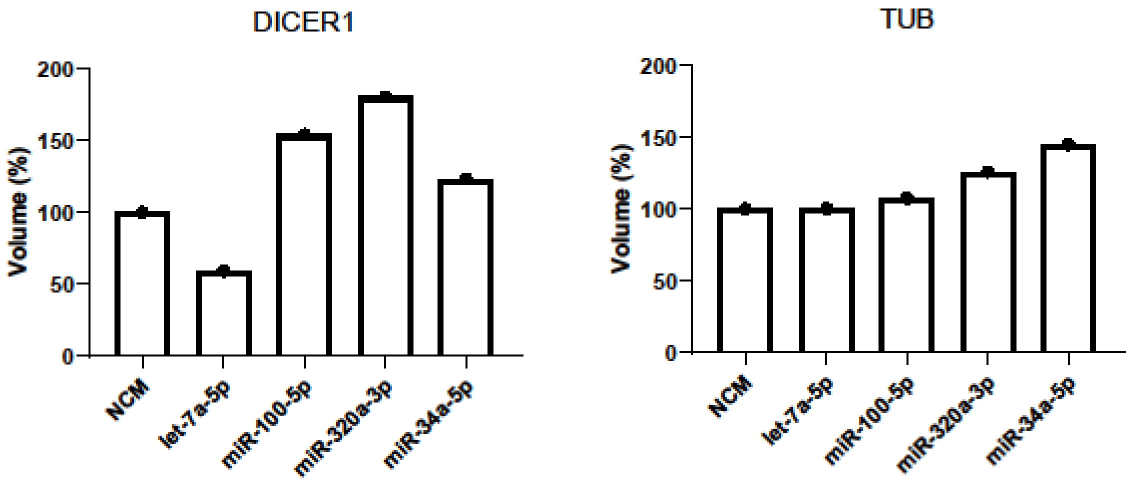

Protein bands have been quantified using the FusionCapt Advance software. The band intensity is expressed as the volume (%) relative to the intensity of the negative control mimic (NCM).

Blot 5

IGR37

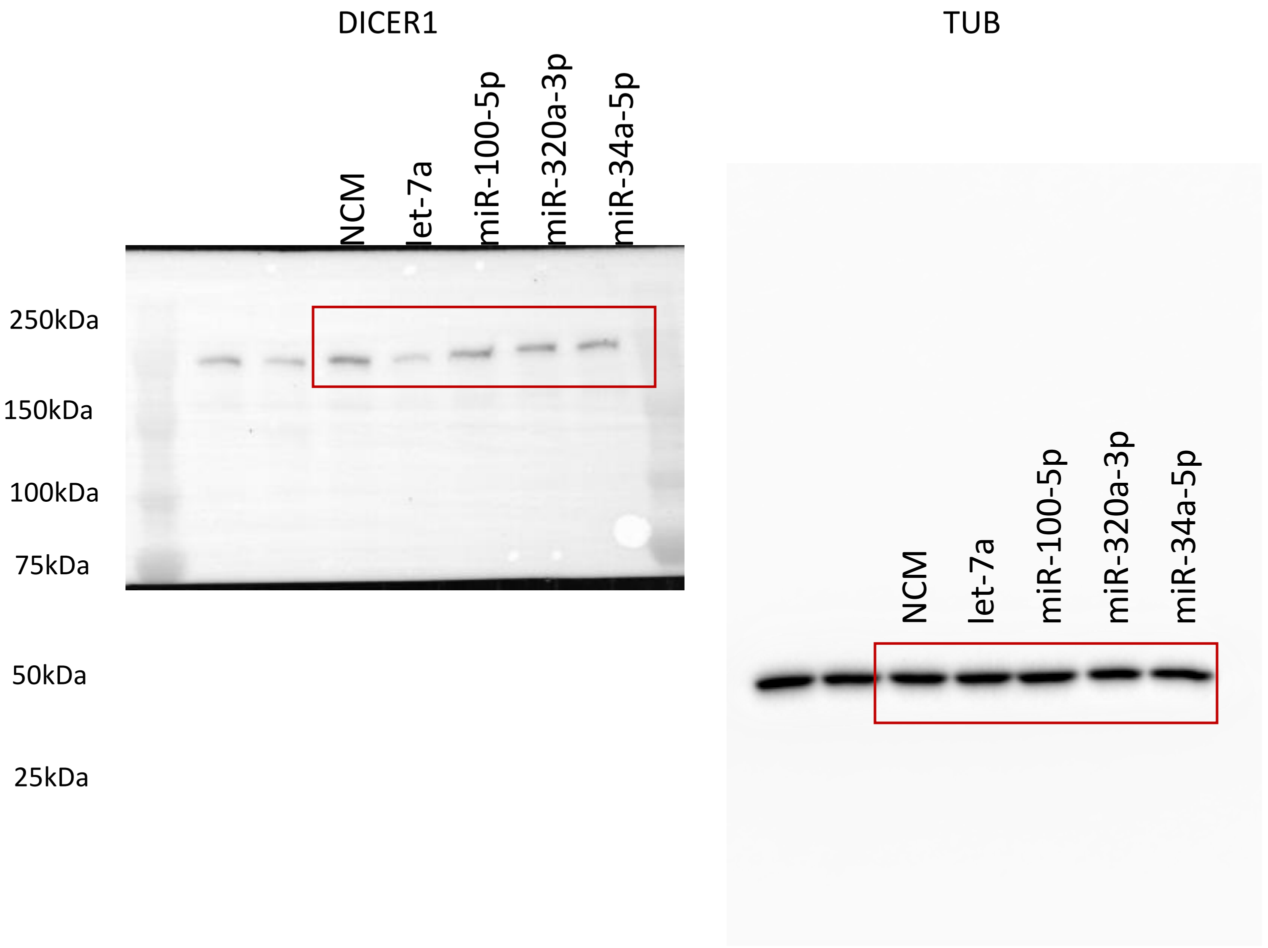

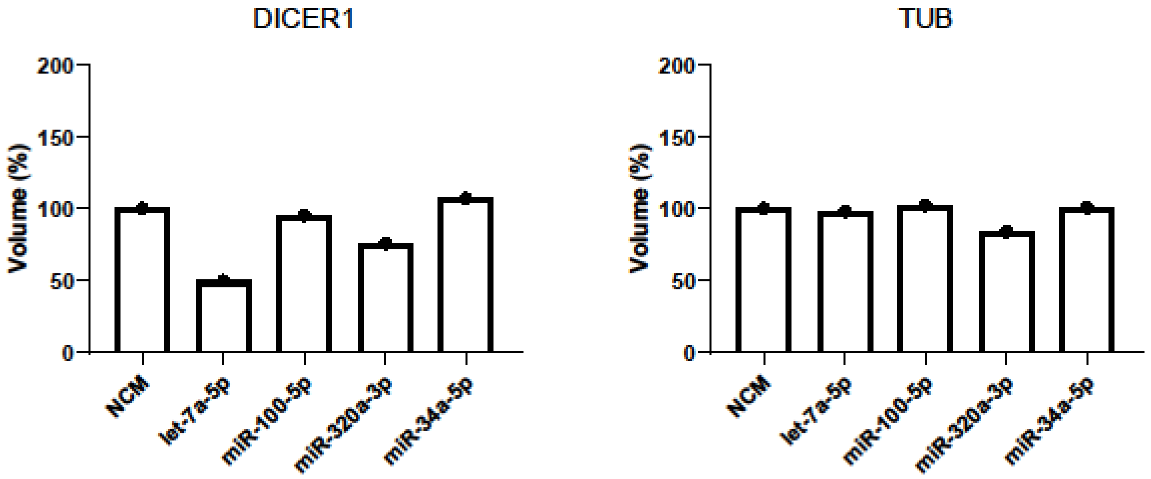

Protein bands have been quantified using the FusionCapt Advance software. The band intensity is expressed as the volume (%) relative to the intensity of the negative control mimic (NCM).

Blot 6

501Mel

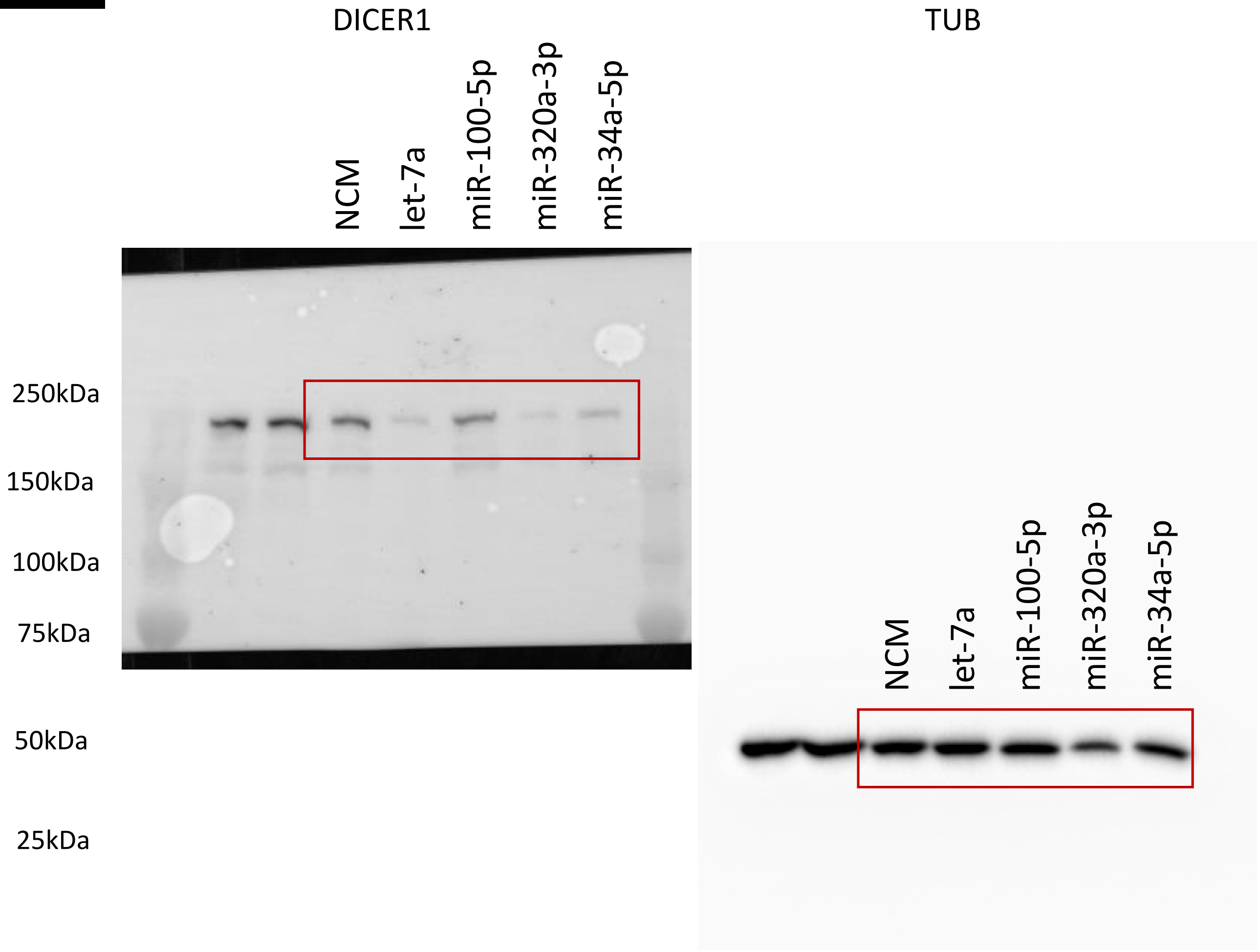

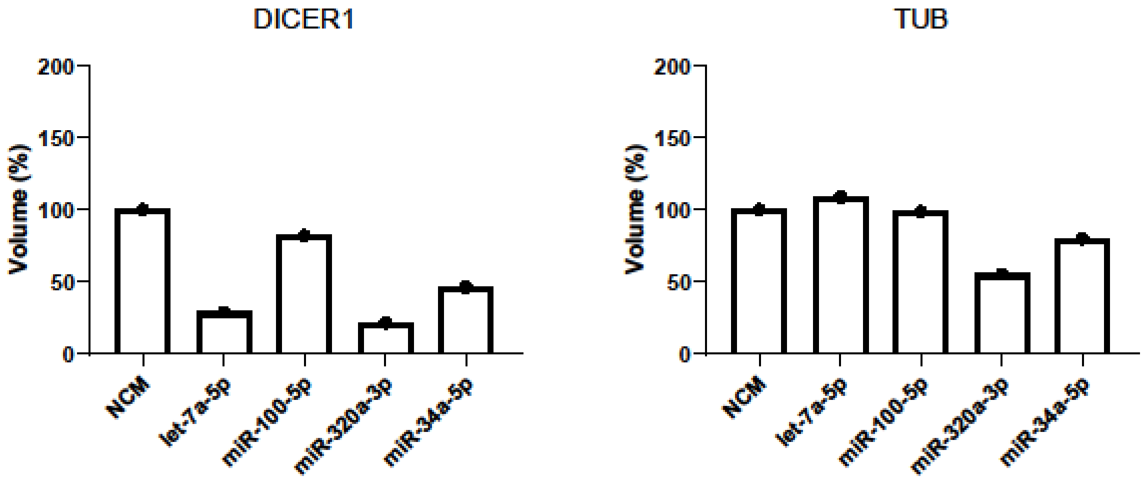

Protein bands have been quantified using the FusionCapt Advance software. The band intensity is expressed as the volume (%) relative to the intensity of the negative control mimic (NCM).
